# National trends in dyslipidemia prevalence, awareness, treatment, and control in South Korea from 2005 to 2022

**DOI:** 10.1038/s41598-025-00354-2

**Published:** 2025-05-09

**Authors:** Hyeseung Lee, Seokjun Kim, Yejun Son, Soeun Kim, Hyeon Jin Kim, Hyesu Jo, Jaeyu Park, Kyeongmin Lee, Hayeon Lee, Jiseung Kang, Selin Woo, Sunyoung Kim, Sang Youl Rhee, Jiyoung Hwang, Lee Smith, Dong Keon Yon

**Affiliations:** 1 Department of Medicine, Kyung Hee University College of Medicine, Seoul, South Korea; 2 Center for Digital Health, Medical Science Research Institute, Kyung Hee University College of Medicine, 23 Kyungheedae-ro, Dongdaemun-gu, Seoul, 02447 South Korea; 3 Department of Precision Medicine, Kyung Hee University College of Medicine, Seoul, South Korea; 4 Department of Regulatory Science, Kyung Hee University, Seoul, South Korea; 5 Department of Electronics and Information Convergence Engineering, Kyung Hee University, Yongin, South Korea; 6 Division of Sleep Medicine, Harvard Medical School, Boston, MA USA; 7 Department of Health and Safety Convergence Science, Korea University Graduate School, Seoul, South Korea; 8 Department of Family Medicine, Kyung Hee University Medical Center, Kyung Hee University College of Medicine, Seoul, South Korea; 9 Department of Endocrinology and Metabolism, Kyung Hee University School of Medicine, Seoul, South Korea; 10 Centre for Health, Performance and Wellbeing, Anglia Ruskin University, East Rd, Cambridge, CB1 1PT UK; 11 Department of Pediatrics, College of Medicine, Kyung Hee University Medical Center, Kyung Hee University, 23 Kyungheedae-ro, Dongdaemun-gu, Seoul, 02447 South Korea

**Keywords:** COVID-19, Pandemic, Dyslipidemia, Prevalence, South Korea, Trend, Dyslipidaemias, Disease prevention

## Abstract

Dyslipidemia has steadily increased in South Korea over the past two decades, emerging as a major public health concern and key risk factor for cardiovascular disease. Thus, our study aimed to investigate long-term trends in the prevalence, awareness, treatment, and control of dyslipidemia in South Korea, including the COVID-19 pandemic. This study utilized data from nationally representative cross-sectional surveys conducted as part of the Korea National Health and Nutrition Examination Survey from 2005 to 2022, analyzing long-term trends of dyslipidemia among 98,396 individuals aged over 30. Weighted linear and binary logistic regression were performed to calculate the β coefficients, β_diff_, and weighted odds ratios with 95% confidence intervals (CIs). Weighted odds ratios were computed for various socioeconomic groups using aggregated data from 2005 to 2022. The prevalence of dyslipidemia increased from 41.30% (95% CI 40.40–42.21) in 2005–2009 to 48.41% (47.36–49.47) in 2020–2022. Awareness increased from 17.87% (16.75–18.99) to 48.90% (47.34–50.47), treatment from 7.10% (6.39–7.80) to 38.19% (36.61–39.76), and control among prevalence from 6.49% (5.79–7.19) to 31.82% (30.33–33.32). Treatment (β_diff_, 3.94 [1.97–5.92]) and control among prevalence (β_diff_, 3.52 [1.67–5.38]) increased more rapidly during the pandemic. Higher odds of dyslipidemia were associated with male sex, older population, rural residence, high BMI, central adiposity, low education and income levels, smoking, and high-risk alcohol consumption. Lower odds of awareness, treatment, and control among individuals with dyslipidemia were associated with male sex, younger population, rural residence, higher education and income levels, smoking, and high-risk alcohol consumption. Over the past 18 years, the prevalence, awareness, treatment, and control of dyslipidemia have steadily increased, with persistent disparities among socioeconomic groups.

**Supplementary Information:**

The online version contains supplementary material available at 10.1038/s41598-025-00354-2.

## Introduction

Cardiovascular diseases (CVD) are one of the leading causes of death and disability worldwide, responsible for approximately 19.7 million deaths and 417 million disability-adjusted life years according to a 2019 study by the World Health Organization^1^. Dyslipidemia, a main cause of CVD, contributes to approximately one-third of ischemic heart disease and one-fifth of cerebrovascular disease globally, resulting in approximately 2.6 million deaths annually^2^. Over the past three decades, the prevalence of dyslipidemia has markedly increased worldwide, making it a significant public health concern^3^.

Since the emergence of the severe acute respiratory syndrome coronavirus 2, there have been numerous global deaths from COVID-19, and many studies have consistently highlighted its negative effects on various diseases^4,5^. Recent research has indicated that as the severity of COVID-19 infection increases, so does the burden of dyslipidemia^6^. Therefore, it is crucial to examine trends in the prevalence of dyslipidemia, including during the pandemic. Previous studies in South Korea have shown a steady rise in the prevalence of dyslipidemia, while treatment rates remain low^7^. Identifying high-risk individuals is essential because early and effective management of dyslipidemia can reduce the burden associated with disease development.

Various studies have been conducted worldwide focusing on the prevalence of dyslipidemia^8,9^. However, to our knowledge, no research has comprehensively examined the trends in prevalence, awareness, treatment, and control of dyslipidemia, including a pre-pandemic and during-pandemic comparison in South Korea. Therefore, this study aimed to investigate the trends of dyslipidemia in South Korea from 2005 to 2022 by category and compare dyslipidemia trends before and during the COVID-19 pandemic. By comparing different sociodemographic groups, this study aimed to identify vulnerable groups for dyslipidemia, thereby contributing to public health.

## Methods

### Survey design and participants

This study analyzed long-term trends in dyslipidemia prevalence, awareness, treatment, and control over 18 years. We used data from the Korea National Health and Nutrition Examination Survey (KNHANES), conducted by the Korea Disease Control and Prevention Agency (KDCA) from 2005 to 2022^10–12^. The KNHANES utilized a robust multistage stratified cluster sampling design, selecting participants from a wide array of geographic and demographic areas to mirror the national profiles of age, sex, region, and socioeconomic status. In addition, sampling weights were applied to correct for differences in selection probability and non-response, thereby improving the representativeness and generalizability of the study outcomes. This study sought to investigate the dynamics of dyslipidemia and identify groups at higher risk by incorporating a broad set of socioeconomic factors.

The participants of this study were aged 30 years and over who were considered at risk for dyslipidemia, following the guidelines established by prior studies in the field^13^. Information of participants in the study covered variables such as age, sex, region of residence, body mass index (BMI), educational background, household income, and smoking status^10,11^. Across the duration of the study, 98,396 individuals were sampled, with yearly breakdowns as follows: 35,005 from 2005 to 2009, 15,610 from 2010 to 2012, 13,440 from 2013 to 2015, 20,601 from 2016 to 2019, and 13,740 from 2020 to 2022, with each segment designed following sampling methodology of KNHANES^14^. Particular focus was placed on 2020–2022 to delve into the effects of the COVID-19 pandemic. The research protocol was approved by the Institutional Review Boards of the KDCA (2007-02CON-04-P, 2008-04EXP-01-C, 2009-01CON-03-2 C, 2010-02CON-21-C, 2011-02CON-06-C, 2012-01EXP-01-2 C, 2013-07CON-03-4 C, 2013-12EXP-035 C). All participants provided written informed consent, and the KNHANES was made publicly available for various epidemiological research efforts. Our study adhered to the ethical standards of the Declaration of Helsinki.

### Health outcomes

In this study, prevalence, awareness, treatment, control among prevalence, and control among treatment were the independent variables. Total cholesterol (TC), high-density lipoprotein cholesterol (HDL-C), low-density lipoprotein cholesterol (LDL-C), and triglycerides (TG) were determined in the assessment of dyslipidemia^13^. Prevalence of dyslipidemia was defined as the proportion of participants with fasting TC ≥ 240 mg/dL, LDL-C ≥ 160 mg/dL, HDL-C < 40 mg/dL, or TG ≥ 200 mg/dL, or who were currently taking lipid-lowering medication or had previously been diagnosed with dyslipidemia by a physician^13^. Awareness was determined as the percentage of patients with dyslipidemia who responded “yes” to the question, “Have you ever been told by a doctor or other healthcare professional that you have dyslipidemia?” Treatment was defined as the proportion of people taking lipid-lowering medication within the prevalence group. Control among prevalence was defined as the proportion of people with TC < 240 mg/dL, LDL-C < 160 mg/dL, HDL-C ≥ 40 mg/dL, and TG < 200 mg/dL within the prevalence group. Control among treatment was defined as the proportion of people taking lipid-lowering medication and meeting the criteria of TC < 240 mg/dL, LDL-C < 160 mg/dL, HDL-C ≥ 40 mg/dL, and TG < 200 mg/dL. The response rates for TC, LDL-C, HDL-C, and TG are presented in Table S1.

### Covariates

In this study, the covariates included sex, age (30–39 years, 40–49 years, 50–59 years, 60–69 years, and ≥ 70 years), region of residence (urban and rural)^15^, BMI group (underweight [< 18.5 kg/m^2^], normal [18.5–22.9 kg/m^2^], overweight [23.0–24.9 kg/m^2^], and obese [≥ 25.0 kg/m^2^]), educational background (elementary school or lower, middle school, high school, and college or higher), household income (lowest, second, third, and highest quartile), smoking status (smoker and non-smoker), waist-to-height ratio (normal and central adiposity), daily calorie intake (low and high), and high-risk drinking (yes and no). The regions of residence of participants were classified as either urban or rural according to their responses in the survey^15^. Household income was segmented into four quartiles derived from the quartiles of standardized income, utilizing sample household and population statistics from the KNHANES. BMI was designated under categories in line with the criteria set by the Asian-Pacific guidelines^15^. Waist-to-height ratio was calculated by dividing waist circumference by height. A ratio of < 0.5 was classified as normal, while ≥ 0.5 indicated central adiposity^16,17^. Daily calorie intake was categorized into two groups, with individuals in the lower 50% of total daily calorie consumption classified as having low intake, while those in the upper 50% were classified as having high intake. High-risk drinking was defined as consuming ≥ 7 drinks per occasion for men or ≥ 5 for women at least twice per week^18,19^. Participants meeting these criteria were classified as “yes”, while others were classified as “no”. These covariates were identified as potential factors influencing the risk of developing dyslipidemia.

### Statistical analysis

Our study implemented a weighted complex sampling method to assess the national prevalence, awareness, treatment, and control of dyslipidemia. Sampling weights were applied to adjust for differential selection probabilities. We used linear and binary logistic regression analyses to estimate β coefficients with 95% confidence intervals (CIs) and weighted odds ratios (wORs) with 95% CIs^20^. To evaluate changes in trends associated with the COVID-19 pandemic, we divided the study period into pre-pandemic (2005–2019) and pandemic (2020–2022) phases. For each phase, weighted linear regression was used to estimate the annual β coefficients for dyslipidemia indicators. The β difference (β_diff_) was then calculated as the difference between the β coefficient during the pandemic and the β coefficient in the pre-pandemic period, thereby quantifying any acceleration, deceleration, or stability in the trends. This approach allows us to directly compare the rates of change across the two periods.

Subgroup analyses were conducted by stratifying participants based on sex, age, region of residence, BMI, educational background, household income, smoking status, waist-to-height ratio, daily calorie intake, and high-risk alcohol consumption. Within these subgroup analyses, the same procedure was applied: β coefficients were estimated for each period and β_diff_ computed to assess how trends varied across different population segments before and during the pandemic. Furthermore, wORs were calculated using combined data from the entire study period to identify demographic and socioeconomic groups with higher prevalence, awareness, treatment, and control rates of dyslipidemia, thus facilitating a comprehensive comparison. The statistical analyses in our study were performed using SAS software (version 9.4, SAS Institute, Cary, NC, USA), employing a two-sided test, with a *P*-value of 0.05 or less deemed to indicate statistical significance^21^.

## Results

**Fig. 1:**
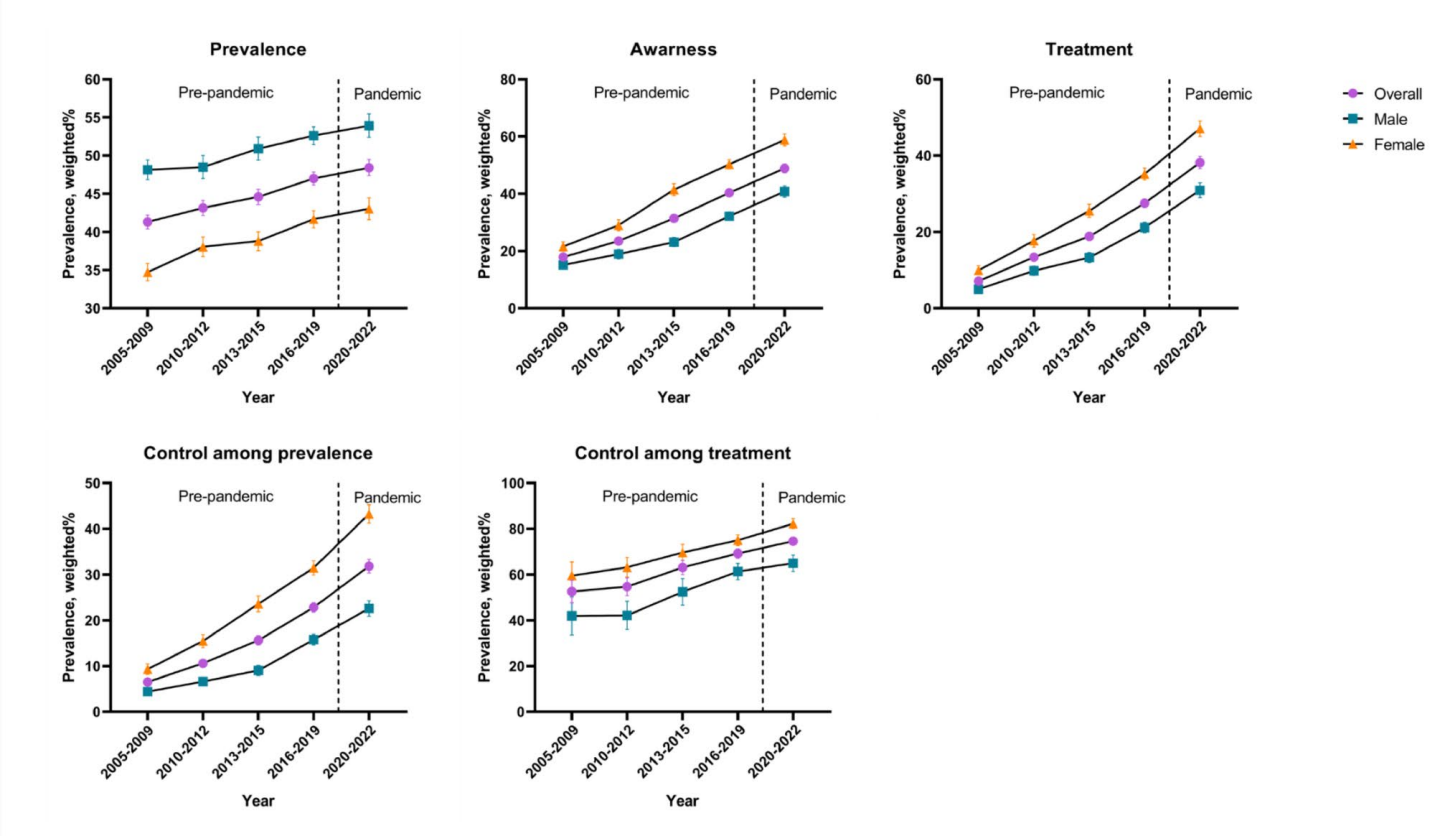
Trends in dyslipidemia prevalence, awareness, treatment, and control rates in population aged ≥ 30 years in South Korea, 2005–2022. The dashed line represents the beginning of the year 2020, marking the onset of the COVID-19 pandemic.

The comprehensive KNHANES survey included 98,396 participants from 2005 to 2022 after handling missing data (male: 48.76%). Table 1 presents the demographic characteristics of the participants. Figure 1 displays the prevalence, awareness, treatment, and control trends of dyslipidemia among individuals aged 30 years and over for different socioeconomic groups over the past 18 years, segmented into periods before and during the COVID-19 pandemic.

**Table 1:** Weighted characteristics of Koreans based on data obtained from the KNHANES from 2005 to 2022 (*n* = 98,396).

	Total	2005–2009	2010–2012	2013–2015	2016–2019	2020–2022
Overall, n (%)	98,396	35,005	15,610	13,440	20,601	13,740
Sex, weighted % (95% CI)						
Male	48.76 (48.44 to 49.08)	49.05 (48.39 to 49.71)	48.68 (47.96 to 49.40)	47.85 (47.08 to 48.63)	48.78 (48.16 to 49.41)	49.36 (48.59 to 50.13)
Female	51.24 (50.92 to 51.56)	50.95 (50.29 to 51.61)	51.32 (50.60 to 52.04)	52.15 (51.37 to 52.93)	51.22 (50.59 to 51.84)	50.64 (49.87 to 51.41)
Age, years, weighted % (95% CI)						
30–39	23.52 (22.95 to 24.09)	28.28 (26.93 to 29.63)	25.87 (24.57 to 27.18)	23.62 (22.29 to 24.94)	21.69 (20.56 to 22.83)	20.01 (18.72 to 21.30)
40–49	25.43 (24.94 to 25.92)	28.31 (27.20 to 29.41)	26.95 (25.73 to 28.16)	25.51 (24.41 to 26.62)	24.48 (23.54 to 25.43)	23.02 (21.83 to 24.21)
50–59	23.20 (22.75 to 23.65)	20.27 (19.40 to 21.15)	22.56 (21.52 to 23.60)	24.10 (23.03 to 25.17)	24.21 (23.34 to 25.08)	23.95 (22.82 to 25.08)
60–69	15.30 (14.95 to 15.65)	12.82 (12.21 to 13.43)	13.14 (12.46 to 13.83)	14.30 (13.54 to 15.05)	16.08 (15.33 to 16.83)	18.98 (18.03 to 19.93)
≥ 70	12.55 (12.21 to 12.90)	10.32 (9.69 to 10.96)	11.48 (10.76 to 12.20)	12.47 (11.69 to 13.25)	13.53 (12.78 to 14.28)	14.04 (13.09 to 14.99)
Region of residence, weighted % (95% CI)						
Urban	81.58 (80.26 to 82.90)	79.13 (76.18 to 82.08)	78.34 (74.90 to 81.79)	81.49 (78.46 to 84.51)	83.83 (81.27 to 86.38)	83.51 (80.51 to 86.51)
Rural	18.42 (17.10 to 19.74)	20.87 (17.92 to 23.82)	21.66 (18.21 to 25.10)	18.51 (15.49 to 21.54)	16.17 (13.62 to 18.73)	16.49 (13.49 to 19.49)
BMI group, weighted % (95% CI)^a^						
Underweight	3.17 (3.02 to 3.32)	3.15 (2.84 to 3.45)	3.22 (2.86 to 3.58)	3.21 (2.86 to 3.55)	3.11 (2.83 to 3.39)	3.19 (2.81 to 3.57)
Normal	36.05 (35.63 to 36.46)	34.52 (33.65 to 35.40)	37.50 (36.48 to 38.51)	37.87 (36.91 to 38.84)	36.45 (35.62 to 37.27)	33.87 (32.89 to 34.86)
Overweight	23.74 (23.38 to 24.10)	23.32 (22.58 to 24.07)	24.28 (23.42 to 25.14)	24.47 (23.63 to 25.31)	23.81 (23.10 to 24.51)	22.87 (22.00 to 23.74)
Obese	35.38 (34.96 to 35.81)	31.72 (30.85 to 32.60)	34.55 (33.54 to 35.56)	34.37 (33.43 to 35.30)	36.29 (35.45 to 37.12)	38.67 (37.59 to 39.76)
Unknown	1.66 (1.58 to 1.75)	7.28 (6.90 to 7.67)	0.45 (0.30 to 0.61)	0.09 (0.02 to 0.15)	0.35 (0.25 to 0.46)	1.40 (1.16 to 1.63)
Educational background, weighted % (95% CI)						
Elementary school or lower	17.26 (16.81 to 17.70)	23.04 (21.98 to 24.10)	20.95 (19.83 to 22.07)	18.11 (17.02 to 19.20)	14.90 (14.02 to 15.79)	11.84 (10.90 to 12.78)
Middle school	10.95 (10.65 to 11.25)	12.98 (12.30 to 13.66)	12.43 (11.71 to 13.15)	11.24 (10.55 to 11.93)	10.18 (9.60 to 10.77)	8.82 (8.16 to 9.48)
High school	31.78 (31.28 to 32.29)	34.17 (33.05 to 35.30)	33.09 (31.90 to 34.29)	32.18 (31.00 to 33.36)	30.14 (29.16 to 31.11)	30.56 (29.40 to 31.72)
College or higher	40.01 (39.28 to 40.73)	29.81 (28.36 to 31.25)	33.53 (31.99 to 35.06)	38.48 (36.90 to 40.05)	44.78 (43.21 to 46.35)	48.78 (47.01 to 50.55)
Household income, weighted % (95% CI)						
Lowest quartile	16.37 (15.89 to 16.85)	17.58 (16.50 to 18.65)	17.50 (16.37 to 18.62)	16.55 (15.37 to 17.72)	16.12 (15.12 to 17.11)	14.64 (13.54 to 15.73)
Second quartile	24.69 (24.16 to 25.21)	24.80 (23.65 to 25.94)	27.51 (26.18 to 28.84)	24.45 (23.21 to 25.69)	24.28 (23.26 to 25.29)	22.86 (21.65 to 24.06)
Third quartile	28.84 (28.31 to 29.38)	28.42 (27.26 to 29.57)	28.11 (26.94 to 29.29)	29.20 (27.83 to 30.58)	28.75 (27.74 to 29.77)	29.63 (28.34 to 30.91)
Highest quartile	30.10 (29.36 to 30.84)	29.21 (27.52 to 30.90)	26.88 (25.44 to 28.32)	29.80 (28.04 to 31.57)	30.86 (29.36 to 32.35)	32.88 (31.02 to 34.74)
Smoking status, weighted % (95% CI)						
Current smoker	21.70 (21.31 to 22.09)	24.16 (23.39 to 24.92)	25.71 (24.75 to 26.68)	22.30 (21.36 to 23.23)	20.33 (19.55 to 21.10)	17.53 (16.64 to 18.42)
Ex-smoker	22.98 (22.64 to 23.32)	20.11 (19.43 to 20.80)	21.31 (20.52 to 22.10)	21.32 (20.53 to 22.10)	23.98 (23.33 to 24.63)	26.83 (25.98 to 27.67)
Non-smoker	54.34 (53.95 to 54.73)	49.70 (48.90 to 50.49)	52.98 (52.11 to 53.85)	56.38 (55.53 to 57.24)	55.69 (54.93 to 56.46)	55.64 (54.62 to 56.66)
Unknown	0.98 (0.91 to 1.04)	6.03 (5.63 to 6.44)	N/A	N/A	N/A	N/A
Waist-to-height ratio, weighted % (95% CI) ^b^						
Normal	46.66 (46.12 to 47.20)	50.89 (49.72 to 52.06)	48.59 (47.30 to 49.87)	49.08 (47.78 to 50.38)	44.65 (43.59 to 45.71)	41.26 (40.00 to 42.53)
Central adiposity	53.34 (52.80 to 53.88)	49.11 (47.94 to 50.28)	51.41 (50.13 to 52.70)	50.92 (49.62 to 52.22)	55.35 (54.29 to 56.41)	58.74 (57.47 to 60.01)
Daily calorie intake, weighted % (95% CI)^c^						
Low	38.59 (38.12 to 39.06)	35.38 (34.44 to 36.31)	39.60 (38.55 to 40.66)	40.02 (38.97 to 41.07)	38.84 (37.93 to 39.74)	38.95 (37.69 to 40.22)
High	46.66 (46.14 to 47.17)	45.29 (44.25 to 46.33)	47.47 (46.32 to 48.61)	48.17 (47.07 to 49.26)	47.27 (46.34 to 48.21)	45.22 (43.79 to 46.64)
Unknown	14.75 (14.26 to 15.25)	19.33 (18.54 to 20.12)	12.93 (12.12 to 13.75)	11.81 (11.04 to 12.59)	13.89 (13.29 to 14.49)	15.83 (14.00 to 17.67)
High-risk drinking, weighted % (95% CI)^d^						
No	85.54 (85.21 to 85.88)	80.94 (80.14 to 81.74)	85.90 (85.07 to 86.73)	86.80 (86.04 to 87.56)	86.94 (86.36 to 87.52)	86.69 (85.90 to 87.49)
Yes	12.91 (12.60 to 13.23)	12.79 (12.12 to 13.46)	13.45 (12.70 to 14.20)	12.21 (11.48 to 12.94)	12.88 (12.31 to 13.45)	13.18 (12.39 to 13.98)
Unknown	1.55 (1.43 to 1.66)	6.27 (5.79 to 6.74)	0.65 (0.38 to 0.93)	0.99 (0.76 to 1.21)	0.18 (0.12 to 0.24)	0.12 (0.05 to 0.20)
Dyslipidemia, weighted % (95% CI)						
Normal	54.76 (54.33 to 55.19)	58.70 (57.79 to 59.60)	56.86 (55.90 to 57.83)	55.40 (54.39 to 56.42)	53.00 (52.15 to 53.84)	51.59 (50.53 to 52.65)
Dyslipidemia	45.24 (44.81 to 45.67)	41.30 (40.40 to 42.21)	43.14 (42.17 to 44.10)	44.60 (43.58 to 45.61)	47.00 (46.16 to 47.85)	48.41 (47.36 to 49.47)

BMI, body mass index; CI confidence interval; KNHANES, Korea National Health and Nutrition Examination Survey.

The table presents the weighted percentage of each socioeconomic group among the total participants.

^a^BMI was divided into four groups according to Asian-Pacific guidelines: underweight (< 18.5 kg/m^2^), normal (18.5–22.9 kg/m^2^), overweight (23.0–24.9 kg/m^2^), and obese (≥ 25 kg/m^2^).

^b^Waist-to-height ratio was calculated as waist circumference divided by height and categorized into two groups: normal (< 0.5) and central adiposity (≥ 0.5).

^c^Daily calorie intake was categorized into two groups: low (below the median) and high (above the median).

^d^High-risk drinking was defined as consuming ≥ 7 drinks per occasion for men or ≥ 5 for women at least twice per week, classified as ‘yes’ or ‘no’ accordingly.

### Prevalence of dyslipidemia

Table 2 shows the trends and differences in the prevalence of dyslipidemia from 2005 to 2022. The weighted prevalence of dyslipidemia increased consistently from 41.30% (95% CI 40.40–42.21) to 48.41% (95% CI 47.36–49.47) between 2005 and 2022. Tables 3 and S3 shows the ORs for the prevalence of dyslipidemia across various socioeconomic groups. Individuals with obesity had significantly higher odds of dyslipidemia (wOR, 5.62 [95% CI 5.01–6.30]). Older population was also significantly associated with increased odds of dyslipidemia (60–69 years: wOR, 3.25; 95% CI 3.07–3.44; ≥70 years: wOR, 2.96; 95% CI 2.81–3.13). Additionally, male sex (wOR, 1.59; 95% CI 1.54–1.65), lower educational background (wOR, 1.95; 95% CI 1.87–2.04]), lower household income (wOR, 1.60; 95% CI 1.52–1.68]), smoking (wOR, 1.46; 95% CI 1.41–1.51]), central adiposity (wOR, 3.14; 95% CI 3.03–3.25]), and high-risk alcohol consumption (wOR, 1.18; 95% CI 1.12–1.25]) were also associated with increased odds of dyslipidemia. As shown in Table S2, the interaction analysis between sex and other covariates revealed that the influence of sex on dyslipidemia prevalence decreased with older population, lower income, and lower education levels, whereas it increased among smokers and high-risk drinkers.

**Table 2:** National trends of the prevalence, awareness, treatment, control among dyslipidemia, and control among treatment before and during the COVID-19 pandemic (weighted % [95% CI]) based on data obtained from the KNHANES.

Variables	Rate	Pre-pandemic				During the pandemic	Trends in the pre-pandemic era, β (95% CI)^a^	Trends in the pandemic era, β (95% CI)^a^	Trend differences, βdiff (95% CI)^a^
		2005–2009	2010–2012	2013–2015	2016–2019	2020–2022			
Overall	Prevalence	41.30 (40.40 to 42.21)	43.14 (42.17 to 44.10)	44.60 (43.58 to 45.61)	47.00 (46.16 to 47.85)	48.41 (47.36 to 49.47)	**1.86 (1.47 to 2.26)**	**1.41 (0.05 to 2.78)**	−0.45 (−1.87 to 0.97)
	Awareness	17.87 (16.75 to 18.99)	23.48 (22.15 to 24.80)	31.42 (30.10 to 32.75)	40.38 (39.19 to 41.57)	48.90 (47.34 to 50.47)	**7.61 (7.08 to 8.13)**	**8.52 (6.55 to 10.50)**	0.92 (−1.13 to 2.96)
	Treatment	7.10 (6.39 to 7.80)	13.37 (12.34 to 14.41)	18.83 (17.72 to 19.95)	27.52 (26.42 to 28.62)	38.19 (36.61 to 39.76)	**6.72 (6.30 to 7.15)**	**10.67 (8.74 to 12.59)**	**3.94 (1.97 to 5.92)**
	Control among prevalence	6.49 (5.79 to 7.19)	10.61 (9.77 to 11.45)	15.62 (14.59 to 16.65)	22.84 (21.82 to 23.86)	31.82 (30.33 to 33.32)	**5.46 (5.07 to 5.86)**	**8.99 (7.17 to 10.80)**	**3.52 (1.67 to 5.38)**
	Control among treatment	52.55 (47.61 to 57.49)	54.68 (50.80 to 58.56)	63.10 (60.04 to 66.16)	69.20 (67.10 to 71.30)	74.55 (72.56 to 76.55)	**6.27 (4.77 to 7.78)**	**5.36 (2.46 to 8.25)**	−0.92 (−4.18 to 2.35)
Sex									
Male	Prevalence	48.14 (46.85 to 49.42)	48.50 (47.00 to 50.00)	50.91 (49.39 to 52.42)	52.61 (51.44 to 53.79)	53.93 (52.39 to 55.47)	**1.59 (1.03 to 2.15)**	1.32 (−0.62 to 3.26)	−0.27 (−2.29 to 1.75)
	Awareness	15.12 (13.67 to 16.57)	18.92 (17.24 to 20.60)	23.11 (21.48 to 24.75)	32.14 (30.61 to 33.67)	40.78 (38.80 to 42.76)	**5.61 (4.94 to 6.29)**	**8.64 (6.13 to 11.14)**	**3.02 (0.43 to 5.62)**
	Treatment	4.96 (4.18 to 5.74)	9.82 (8.64 to 10.99)	13.28 (11.95 to 14.60)	21.14 (19.76 to 22.51)	30.91 (28.98 to 32.84)	**5.26 (4.74 to 5.77)**	**9.77 (7.40 to 12.15)**	**4.52 (2.09 to 6.95)**
	Control among prevalence	4.40 (3.60 to 5.19)	6.62 (5.68 to 7.57)	9.02 (7.90 to 10.14)	15.75 (14.59 to 16.91)	22.58 (20.88 to 24.28)	**3.72 (3.27 to 4.17)**	**6.83 (4.77 to 8.89)**	**3.11 (1.00 to 5.22)**
	Control among treatment	41.91 (33.57 to 50.24)	42.14 (35.96 to 48.32)	52.42 (46.59 to 58.25)	61.29 (57.72 to 64.86)	64.93 (61.37 to 68.49)	**7.83 (5.31 to 10.35)**	3.65 (−1.40 to 8.70)	−4.18 (−9.83 to 1.46)
Female	Prevalence	34.73 (33.59 to 35.87)	38.05 (36.77 to 39.33)	38.80 (37.56 to 40.04)	41.66 (40.54 to 42.78)	43.04 (41.61 to 44.46)	**2.15 (1.64 to 2.66)**	1.38 (−0.46 to 3.22)	−0.77 (−2.68 to 1.13)
	Awareness	21.54 (19.91 to 23.18)	28.99 (27.10 to 30.89)	41.43 (39.36 to 43.50)	50.29 (48.69 to 51.89)	58.83 (56.78 to 60.87)	**9.90 (9.16 to 10.63)**	**8.54 (5.93 to 11.14)**	−1.36 (−4.06 to 1.35)
	Treatment	9.95 (8.78 to 11.12)	17.68 (16.01 to 19.34)	25.52 (23.81 to 27.24)	35.20 (33.67 to 36.73)	47.07 (45.01 to 49.13)	**8.41 (7.77 to 9.04)**	**11.87 (9.31 to 14.43)**	**3.47 (0.83 to 6.11)**
	Control among prevalence	9.30 (8.14 to 10.46)	15.44 (14.01 to 16.87)	23.58 (21.86 to 25.31)	31.46 (29.92 to 33.01)	43.21 (41.19 to 45.22)	**7.50 (6.88 to 8.13)**	**11.74 (9.20 to 14.28)**	**4.24 (1.62 to 6.86)**
	Control among treatment	59.54 (53.58 to 65.49)	63.15 (58.85 to 67.45)	69.66 (66.23 to 73.09)	74.95 (72.60 to 77.30)	82.21 (80.06 to 84.36)	**5.46 (3.73 to 7.18)**	**7.26 (4.07 to 10.44)**	1.80 (−1.83 to 5.43)
Age, years									
30–39	Prevalence	31.37 (29.72 to 33.02)	30.45 (28.52 to 32.38)	30.83 (28.71 to 32.94)	30.42 (28.79 to 32.05)	30.24 (27.93 to 32.55)	−0.25 (−1.01 to 0.50)	−0.18 (−3.01 to 2.65)	0.07 (−2.86 to 3.00)
	Awareness	8.25 (6.34 to 10.16)	8.21 (5.92 to 10.51)	10.42 (7.99 to 12.85)	10.46 (8.41 to 12.51)	11.53 (8.73 to 14.34)	0.88 (−0.03 to 1.78)	1.07 (−2.40 to 4.54)	0.20 (−3.39 to 3.78)
	Treatment	1.46 (0.65 to 2.26)	2.02 (0.85 to 3.19)	3.02 (1.77 to 4.26)	3.56 (2.40 to 4.71)	6.36 (4.24 to 8.47)	**0.73 (0.28 to 1.18)**	**2.80 (0.39 to 5.21)**	2.07 (−0.38 to 4.52)
	Control among prevalence	2.25 (1.26 to 3.23)	2.52 (1.31 to 3.72)	3.40 (2.01 to 4.78)	4.79 (3.43 to 6.15)	5.90 (3.71 to 8.09)	**0.84 (0.31 to 1.37)**	1.12 (−1.46 to 3.69)	0.28 (−2.35 to 2.91)
	Control among treatment	33.13 (5.79 to 60.47)	34.00 (6.44 to 61.56)	34.59 (13.95 to 55.22)	59.91 (43.47 to 76.35)	60.07 (42.38 to 77.76)	9.24 (−0.81 to 19.30)	0.16 (−24.07 to 24.39)	−9.08 (−35.32 to 17.15)
40–49	Prevalence	37.99 (36.31 to 39.66)	37.92 (35.96 to 39.87)	37.48 (35.51 to 39.46)	40.26 (38.69 to 41.84)	41.81 (39.82 to 43.80)	0.64 (−0.10 to 1.39)	1.55 (−0.98 to 4.08)	0.91 (−1.73 to 3.54)
	Awareness	15.19 (13.16 to 17.22)	14.80 (12.38 to 17.23)	20.95 (18.09 to 23.81)	24.83 (22.34 to 27.33)	31.93 (28.75 to 35.11)	**3.51 (2.47 to 4.54)**	**7.10 (3.06 to 11.14)**	3.59 (−0.58 to 7.76)
	Treatment	3.79 (2.74 to 4.85)	5.47 (3.98 to 6.95)	9.80 (7.73 to 11.87)	12.54 (10.49 to 14.59)	21.12 (18.27 to 23.97)	**3.05 (2.31 to 3.80)**	**8.58 (5.07 to 12.09)**	**5.53 (1.94 to 9.12)**
	Control among prevalence	4.75 (3.51 to 5.98)	5.50 (3.97 to 7.04)	8.76 (6.76 to 10.76)	11.28 (9.55 to 13.01)	17.32 (14.75 to 19.90)	**2.29 (1.60 to 2.97)**	**6.04 (2.93 to 9.15)**	**3.75 (0.57 to 6.93)**
	Control among treatment	46.53 (32.66 to 60.39)	52.67 (37.86 to 67.48)	53.88 (42.18 to 65.58)	66.23 (57.54 to 74.92)	67.30 (60.58 to 74.03)	**6.66 (1.47 to 11.84)**	1.07 (−9.90 to 12.05)	−5.58 (−17.72 to 6.56)
50–59	Prevalence	47.46 (45.53 to 49.39)	50.41 (48.36 to 52.47)	49.99 (48.00 to 51.97)	52.86 (51.13 to 54.59)	54.13 (51.94 to 56.32)	**1.57 (0.75 to 2.40)**	1.27 (−1.52 to 4.06)	−0.30 (−3.21 to 2.61)
	Awareness	26.74 (24.06 to 29.41)	29.18 (26.48 to 31.89)	34.33 (31.70 to 36.96)	43.83 (41.66 to 46.00)	49.91 (46.92 to 52.89)	**5.85 (4.77 to 6.94)**	**6.08 (2.40 to 9.77)**	0.23 (−3.61 to 4.07)
	Treatment	10.34 (8.61 to 12.06)	16.53 (14.39 to 18.68)	18.67 (16.42 to 20.92)	28.17 (26.09 to 30.25)	37.01 (34.08 to 39.94)	**5.66 (4.77 to 6.55)**	**8.84 (5.26 to 12.43)**	3.18 (−0.51 to 6.88)
	Control among prevalence	9.64 (7.98 to 11.29)	12.27 (10.61 to 13.94)	16.06 (13.93 to 18.18)	22.91 (20.98 to 24.85)	29.89 (27.06 to 32.73)	**4.49 (3.67 to 5.31)**	**6.98 (3.55 to 10.42)**	2.49 (−1.04 to 6.03)
	Control among treatment	50.63 (41.67 to 59.58)	51.04 (43.83 to 58.25)	62.60 (55.95 to 69.25)	66.43 (62.00 to 70.87)	72.63 (68.16 to 77.11)	**6.24 (3.32 to 9.15)**	6.20 (−0.12 to 12.52)	−0.03 (−6.99 to 6.92)
60–69	Prevalence	53.06 (51.02 to 55.10)	57.01 (54.73 to 59.29)	57.86 (55.70 to 60.01)	61.90 (60.07 to 63.73)	60.66 (58.52 to 62.81)	**2.76 (1.88 to 3.64)**	−1.23 (−4.05 to 1.59)	**−3.99 (−6.94 to −1.04)**
	Awareness	25.18 (22.45 to 27.90)	38.84 (36.02 to 41.65)	51.44 (48.60 to 54.28)	58.34 (56.02 to 60.65)	64.88 (62.14 to 67.62)	**10.96 (9.82 to 12.10)**	**6.55 (2.94 to 10.15)**	**−4.41 (−8.19 to −0.63)**
	Treatment	13.82 (11.84 to 15.79)	26.53 (23.97 to 29.08)	33.89 (31.27 to 36.50)	43.63 (41.38 to 45.87)	53.00 (50.25 to 55.75)	**9.60 (8.61 to 10.58)**	**9.37 (5.81 to 12.94)**	−0.22 (−3.92 to 3.47)
	Control among prevalence	11.14 (9.18 to 13.10)	20.79 (18.49 to 23.08)	28.46 (25.82 to 31.10)	35.45 (33.08 to 37.83)	46.03 (43.27 to 48.80)	**7.97 (6.97 to 8.98)**	**10.58 (6.92 to 14.24)**	2.61 (−1.19 to 6.40)
	Control among treatment	59.30 (51.68 to 66.91)	57.60 (51.44 to 63.76)	67.20 (62.87 to 71.53)	69.33 (66.11 to 72.55)	77.45 (74.26 to 80.64)	**4.41 (2.04 to 6.77)**	**8.12 (3.59 to 12.65)**	3.71 (−1.40 to 8.82)
≥ 70	Prevalence	50.92 (48.73 to 53.12)	53.79 (51.46 to 56.12)	59.60 (57.41 to 61.79)	57.60 (55.73 to 59.47)	58.83 (56.77 to 60.89)	**2.44 (1.52 to 3.36)**	1.23 (−1.54 to 4.01)	−1.20 (−4.13 to 1.72)
	Awareness	13.90 (11.75 to 16.04)	28.16 (24.99 to 31.34)	38.49 (35.37 to 41.61)	56.79 (54.19 to 59.39)	72.21 (69.63 to 74.78)	**14.05 (12.91 to 15.19)**	**15.42 (11.75 to 19.08)**	1.37 (−2.46 to 5.21)
	Treatment	8.74 (6.98 to 10.50)	19.16 (16.36 to 21.96)	29.48 (26.56 to 32.40)	45.12 (42.63 to 47.62)	62.58 (59.75 to 65.40)	**12.13 (11.09 to 13.17)**	**17.45 (13.69 to 21.22)**	**5.32 (1.41 to 9.23)**
	Control among prevalence	5.56 (4.08 to 7.04)	14.15 (11.75 to 16.55)	21.76 (19.21 to 24.30)	37.25 (34.77 to 39.73)	52.12 (49.24 to 55.00)	**10.50 (9.53 to 11.48)**	**14.87 (11.07 to 18.67)**	**4.36 (0.44 to 8.29)**
	Control among treatment	54.09 (43.01 to 65.16)	59.46 (52.81 to 66.12)	65.15 (59.89 to 70.42)	73.66 (70.45 to 76.87)	76.86 (73.84 to 79.88)	**6.95 (4.20 to 9.70)**	3.20 (−1.22 to 7.62)	−3.76 (−8.96 to 1.45)
Region of residence									
Urban	Prevalence	40.80 (39.78 to 41.81)	42.55 (41.46 to 43.64)	43.50 (42.35 to 44.65)	46.46 (45.55 to 47.38)	47.90 (46.72 to 49.08)	**1.82 (1.38 to 2.25)**	1.44 (−0.07 to 2.94)	−0.38 (−1.95 to 1.19)
	Awareness	18.66 (17.33 to 19.98)	24.32 (22.78 to 25.86)	32.22 (30.76 to 33.69)	40.10 (38.77 to 41.42)	48.84 (47.08 to 50.61)	**7.27 (6.67 to 7.87)**	**8.75 (6.54 to 10.96)**	1.48 (−0.81 to 3.77)
	Treatment	7.35 (6.52 to 8.18)	13.98 (12.81 to 15.15)	19.68 (18.42 to 20.95)	27.13 (25.92 to 28.35)	37.97 (36.18 to 39.75)	**6.53 (6.05 to 7.01)**	**10.83 (8.68 to 12.99)**	**4.31 (2.09 to 6.52)**
	Control among prevalence	6.94 (6.10 to 7.77)	11.08 (10.10 to 12.06)	16.35 (15.17 to 17.53)	22.64 (21.53 to 23.74)	31.95 (30.24 to 33.67)	**5.28 (4.84 to 5.73)**	**9.32 (7.28 to 11.35)**	**4.03 (1.95 to 6.12)**
	Control among treatment	52.65 (47.16 to 58.14)	55.25 (51.08 to 59.41)	63.92 (60.46 to 67.39)	69.63 (67.32 to 71.94)	75.34 (73.06 to 77.61)	**6.30 (4.64 to 7.95)**	**5.71 (2.46 to 8.95)**	−0.59 (−4.23 to 3.05)
Rural	Prevalence	43.23 (41.24 to 45.23)	45.26 (43.15 to 47.36)	49.42 (47.04 to 51.79)	49.79 (47.61 to 51.97)	51.02 (48.53 to 53.52)	**2.40 (1.46 to 3.34)**	1.23 (−2.09 to 4.55)	−1.17 (−4.62 to 2.28)
	Awareness	15.06 (13.24 to 16.88)	20.61 (18.10 to 23.13)	28.32 (25.22 to 31.43)	41.74 (38.94 to 44.54)	49.19 (45.87 to 52.51)	**8.79 (7.71 to 9.88)**	**7.45 (3.08 to 11.81)**	−1.34 (−5.84 to 3.15)
	Treatment	6.18 (4.96 to 7.40)	11.31 (9.18 to 13.44)	15.56 (13.33 to 17.78)	29.39 (26.69 to 32.10)	39.22 (35.90 to 42.55)	**7.39 (6.43 to 8.36)**	**9.83 (5.51 to 14.15)**	2.44 (−1.99 to 6.86)
	Control among prevalence	4.91 (3.83 to 5.99)	9.02 (7.45 to 10.60)	12.81 (10.84 to 14.78)	23.81 (21.10 to 26.52)	31.21 (28.17 to 34.24)	**6.05 (5.13 to 6.96)**	**7.39 (3.29 to 11.50)**	1.35 (−2.85 to 5.55)
	Control among treatment	52.11 (40.76 to 63.46)	52.29 (42.18 to 62.39)	59.18 (52.94 to 65.41)	67.25 (62.05 to 72.44)	70.95 (66.84 to 75.07)	**6.11 (2.42 to 9.80)**	3.71 (−2.93 to 10.34)	−2.40 (−10.00 to 5.19)
BMI group^b^									
Underweight	Prevalence	18.70 (15.03 to 22.37)	21.50 (17.16 to 25.84)	21.31 (17.21 to 25.40)	19.04 (15.73 to 22.36)	22.39 (17.77 to 27.02)	0.04 (−1.56 to 1.63)	3.35 (−2.36 to 9.05)	3.31 (−2.61 to 9.23)
	Awareness	7.50 (2.87 to 12.12)	15.51 (8.06 to 22.97)	19.38 (11.84 to 26.91)	31.15 (22.08 to 40.22)	49.21 (37.72 to 60.69)	**7.48 (4.18 to 10.78)**	**18.06 (3.42 to 32.69)**	10.57 (−4.43 to 25.58)
	Treatment	1.94 (0.00 to 3.87)	9.01 (3.04 to 14.98)	13.36 (6.63 to 20.10)	13.80 (8.07 to 19.54)	36.20 (24.94 to 47.45)	**3.92 (1.82 to 6.03)**	**22.39 (9.75 to 35.04)**	**18.47 (5.65 to 31.29)**
	Control among prevalence	3.84 (0.14 to 7.53)	6.85 (2.29 to 11.41)	13.78 (7.14 to 20.43)	24.27 (16.00 to 32.53)	41.20 (29.36 to 53.03)	**6.91 (4.04 to 9.78)**	**16.93 (2.50 to 31.36)**	10.02 (−4.70 to 24.74)
	Control among treatment	72.46 (26.68 to 100.00)	39.05 (10.56 to 67.55)	73.95 (48.00 to 99.90)	84.72 (68.05 to 100.00)	86.72 (74.16 to 99.28)	**15.94 (1.52 to 30.36)**	2.00 (−18.89 to 22.90)	−13.94 (−39.33 to 11.45)
Normal	Prevalence	31.59 (30.27 to 32.91)	31.12 (29.69 to 32.56)	32.52 (31.01 to 34.03)	34.56 (33.32 to 35.80)	35.15 (33.42 to 36.87)	**1.07 (0.48 to 1.65)**	0.59 (−1.55 to 2.73)	−0.47 (−2.69 to 1.74)
	Awareness	15.74 (13.70 to 17.79)	20.65 (18.47 to 22.82)	30.97 (28.49 to 33.46)	39.22 (37.11 to 41.33)	50.15 (47.36 to 52.94)	**8.13 (7.19 to 9.08)**	**10.93 (7.42 to 14.44)**	2.80 (−0.84 to 6.43)
	Treatment	5.27 (4.18 to 6.37)	11.28 (9.40 to 13.15)	17.96 (15.85 to 20.08)	25.93 (24.09 to 27.77)	38.19 (35.44 to 40.94)	**6.90 (6.19 to 7.62)**	**12.26 (8.94 to 15.58)**	**5.36 (1.97 to 8.75)**
	Control among prevalence	6.57 (5.27 to 7.88)	11.07 (9.42 to 12.72)	18.77 (16.64 to 20.90)	24.56 (22.76 to 26.37)	36.62 (33.81 to 39.43)	**6.19 (5.46 to 6.92)**	**12.06 (8.71 to 15.40)**	**5.87 (2.44 to 9.29)**
	Control among treatment	60.74 (50.51 to 70.96)	67.87 (60.65 to 75.08)	71.74 (65.75 to 77.73)	76.62 (73.06 to 80.19)	83.94 (80.81 to 87.07)	**4.86 (2.02 to 7.71)**	**7.32 (2.57 to 12.07)**	2.46 (−3.07 to 7.99)
Overweight	Prevalence	46.66 (44.88 to 48.44)	44.68 (42.66 to 46.69)	47.40 (45.43 to 49.37)	49.12 (47.45 to 50.79)	50.60 (48.60 to 52.61)	**1.05 (0.27 to 1.84)**	1.48 (−1.13 to 4.10)	0.43 (−2.30 to 3.16)
	Awareness	16.27 (14.31 to 18.23)	24.23 (21.74 to 26.71)	32.51 (29.92 to 35.10)	40.81 (38.52 to 43.09)	50.47 (47.53 to 53.41)	**8.19 (7.22 to 9.17)**	**9.66 (5.93 to 13.40)**	1.47 (−2.39 to 5.33)
	Treatment	6.59 (5.36 to 7.82)	12.90 (11.09 to 14.71)	18.95 (16.85 to 21.05)	27.27 (25.26 to 29.28)	40.08 (37.27 to 42.88)	**6.84 (6.07 to 7.61)**	**12.81 (9.35 to 16.26)**	**5.97 (2.43 to 9.51)**
	Control among prevalence	6.40 (5.14 to 7.66)	11.82 (9.99 to 13.65)	15.51 (13.62 to 17.40)	22.51 (20.64 to 24.38)	34.89 (32.06 to 37.72)	**5.23 (4.50 to 5.96)**	**12.38 (8.98 to 15.78)**	**7.15 (3.67 to 10.62)**
	Control among treatment	55.13 (45.67 to 64.60)	58.91 (50.91 to 66.92)	62.60 (56.73 to 68.48)	68.05 (64.22 to 71.87)	79.52 (76.12 to 82.93)	**4.44 (1.54 to 7.33)**	**11.48 (6.37 to 16.59)**	**7.04 (1.17 to 12.92)**
Obese	Prevalence	58.22 (56.52 to 59.91)	57.24 (55.57 to 58.92)	58.02 (56.26 to 59.78)	60.53 (59.20 to 61.86)	60.82 (59.24 to 62.39)	**0.84 (0.16 to 1.53)**	0.28 (−1.78 to 2.35)	−0.56 (−2.74 to 1.62)
	Awareness	19.41 (17.65 to 21.18)	25.05 (23.10 to 27.00)	31.49 (29.46 to 33.53)	41.01 (39.36 to 42.66)	47.15 (44.95 to 49.34)	**7.21 (6.44 to 7.99)**	**6.13 (3.38 to 8.89)**	−1.08 (−3.94 to 1.78)
	Treatment	8.27 (7.15 to 9.38)	15.03 (13.45 to 16.61)	19.51 (17.84 to 21.18)	28.85 (27.24 to 30.45)	36.91 (34.80 to 39.03)	**6.69 (6.04 to 7.34)**	**8.07 (5.40 to 10.73)**	1.38 (−1.36 to 4.12)
	Control among prevalence	6.60 (5.59 to 7.60)	9.80 (8.58 to 11.01)	13.79 (12.37 to 15.20)	21.94 (20.49 to 23.39)	27.44 (25.49 to 29.40)	**5.11 (4.54 to 5.68)**	**5.50 (3.07 to 7.94)**	0.39 (−2.11 to 2.89)
	Control among treatment	48.58 (41.49 to 55.67)	46.88 (41.27 to 52.50)	57.94 (53.61 to 62.26)	65.70 (62.67 to 68.73)	66.88 (63.74 to 70.02)	**7.22 (5.05 to 9.40)**	1.18 (−3.18 to 5.54)	**−6.04 (−10.91 to −1.17)**
Educational background									
Elementary school or lower	Prevalence	48.37 (46.74 to 50.01)	54.16 (52.19 to 56.12)	59.19 (57.19 to 61.20)	59.47 (57.57 to 61.37)	59.84 (57.42 to 62.25)	**3.94 (3.13 to 4.74)**	0.37 (−2.71 to 3.45)	**−3.57 (−6.75 to −0.39)**
	Awareness	18.16 (16.20 to 20.12)	28.86 (26.29 to 31.44)	39.65 (36.97 to 42.32)	57.05 (54.57 to 59.54)	67.88 (64.89 to 70.87)	**12.69 (11.68 to 13.71)**	**10.83 (6.92 to 14.73)**	−1.87 (−−5.91 to 2.17)
	Treatment	9.62 (8.24 to 11.00)	20.02 (17.63 to 22.42)	26.92 (24.61 to 29.23)	42.49 (40.18 to 44.80)	58.56 (55.58 to 61.54)	**10.50 (9.63 to 11.36)**	**16.07 (12.28 to 19.87)**	**5.58 (1.69 to 9.47)**
	Control among prevalence	6.59 (5.47 to 7.72)	15.12 (13.19 to 17.04)	20.86 (18.75 to 22.96)	33.76 (31.35 to 36.16)	48.07 (44.82 to 51.31)	**8.67 (7.84 to 9.50)**	**14.31 (10.27 to 18.35)**	**5.64 (1.52 to 9.77)**
	Control among treatment	50.79 (43.60 to 57.97)	57.82 (52.23 to 63.42)	62.71 (57.87 to 67.56)	68.60 (65.13 to 72.07)	76.03 (72.46 to 79.59)	**5.70 (3.40 to 8.00)**	**7.43 (2.45 to 12.40)**	1.73 (−3.75 to 7.21)
Middle school	Prevalence	43.26 (40.91 to 45.61)	49.50 (46.66 to 52.33)	53.81 (50.97 to 56.66)	58.20 (55.78 to 60.62)	58.48 (55.61 to 61.35)	**4.93 (3.84 to 6.01)**	0.28 (-3.48 to 4.04)	**−4.65 (−8.56 to −0.74)**
	Awareness	20.77 (18.06 to 23.48)	32.52 (29.01 to 36.03)	41.91 (38.24 to 45.57)	53.11 (50.10 to 56.11)	65.65 (61.85 to 69.45)	**10.63 (9.31 to 11.95)**	**12.54 (7.71 to 17.38)**	1.92 (−3.09 to 6.92)
	Treatment	10.42 (8.34 to 12.51)	18.86 (16.03 to 21.68)	24.01 (20.98 to 27.03)	39.08 (36.05 to 42.12)	53.95 (50.12 to 57.78)	**9.20 (8.00 to 10.40)**	**14.87 (9.99 to 19.75)**	**5.67 (0.64 to 10.69)**
	Control among prevalence	8.59 (6.63 to 10.56)	15.39 (12.73 to 18.04)	21.21 (18.15 to 24.27)	31.54 (28.83 to 34.25)	46.22 (42.37 to 50.07)	**7.51 (6.42 to 8.60)**	**14.68 (9.97 to 19.39)**	**7.17 (2.33 to 12.00)**
	Control among treatment	58.82 (47.77 to 69.86)	55.40 (46.16 to 64.64)	67.21 (60.38 to 74.04)	68.64 (64.07 to 73.22)	75.88 (71.53 to 80.22)	**4.63 (1.22 to 8.05)**	**7.23 (0.92 to 13.54)**	2.60 (−4.57 to 9.78)
High school	Prevalence	38.33 (36.75 to 39.91)	39.78 (38.00 to 41.56)	42.44 (40.66 to 44.22)	47.65 (46.16 to 49.15)	50.93 (48.95 to 52.91)	**3.07 (2.37 to 3.77)**	**3.27 (0.79 to 5.75)**	0.20 (−2.38 to 2.78)
	Awareness	17.11 (15.18 to 19.05)	21.13 (18.97 to 23.29)	30.11 (27.78 to 32.44)	39.49 (37.53 to 41.45)	50.65 (48.07 to 53.24)	**7.69 (6.80 to 8.57)**	**11.17 (7.91 to 14.42)**	**3.48 (0.11 to 6.85)**
	Treatment	5.19 (4.06 to 6.33)	10.93 (9.31 to 12.55)	17.32 (15.34 to 19.30)	26.95 (25.12 to 28.77)	38.91 (36.22 to 41.61)	**7.23 (6.53 to 7.93)**	**11.97 (8.71 to 15.23)**	**4.74 (1.40 to 8.07)**
	Control among prevalence	6.41 (5.13 to 7.68)	9.15 (7.77 to 10.53)	15.02 (13.30 to 16.74)	22.12 (20.44 to 23.80)	31.76 (29.31 to 34.20)	**5.36 (4.69 to 6.04)**	**9.64 (6.67 to 12.60)**	**4.27 (1.23 to 7.31)**
	Control among treatment	52.72 (42.21 to 63.23)	56.91 (49.51 to 64.31)	63.29 (57.27 to 69.32)	69.03 (65.41 to 72.65)	73.40 (69.79 to 77.01)	**5.71 (2.80 to 8.63)**	4.37 (-0.74 to 9.48)	−1.34 (−7.23 to 4.54)
College or higher	Prevalence	38.40 (36.71 to 40.10)	37.21 (35.48 to 38.93)	36.84 (35.21 to 38.46)	39.87 (38.68 to 41.05)	42.25 (40.85 to 43.65)	0.56 (−0.09 to 1.21)	**2.38 (0.54 to 4.22)**	1.82 (−0.13 to 3.77)
	Awareness	17.03 (14.85 to 19.21)	16.60 (14.44 to 18.76)	22.01 (19.88 to 24.13)	28.60 (26.74 to 30.45)	36.87 (34.59 to 39.15)	**4.26 (3.35 to 5.17)**	**8.27 (5.33 to 11.22)**	**4.01 (0.93 to 7.10)**
	Treatment	5.19 (3.93 to 6.44)	7.20 (5.84 to 8.55)	11.98 (10.39 to 13.56)	16.71 (15.14 to 18.29)	26.69 (24.52 to 28.86)	**4.03 (3.37 to 4.69)**	**9.97 (7.28 to 12.67)**	**5.95 (3.17 to 8.72)**
	Control among prevalence	5.47 (4.15 to 6.79)	5.73 (4.47 to 6.98)	9.96 (8.44 to 11.48)	15.30 (13.88 to 16.72)	22.93 (20.83 to 25.03)	**3.56 (2.93 to 4.19)**	**7.63 (5.09 to 10.17)**	**4.07 (1.45 to 6.69)**
	Control among treatment	48.97 (36.88 to 61.05)	42.35 (33.38 to 51.32)	60.00 (52.74 to 67.27)	70.57 (66.21 to 74.93)	74.06 (70.03 to 78.09)	**9.87 (6.36 to 13.38)**	3.49 (−2.46 to 9.45)	−6.37 (−13.29 to 0.54)
Household income									
Lowest quartile	Prevalence	47.04 (45.01 to 49.06)	51.26 (49.08 to 53.44)	57.39 (55.21 to 59.57)	56.14 (54.25 to 58.03)	57.66 (55.27 to 60.04)	**3.31 (2.42 to 4.20)**	1.52 (−1.53 to 4.57)	−1.79 (−4.97 to 1.38)
	Awareness	17.28 (14.92 to 19.65)	26.20 (23.51 to 28.89)	36.28 (33.49 to 39.07)	52.09 (49.54 to 54.64)	61.60 (58.31 to 64.88)	**11.55 (10.44 to 12.67)**	**9.51 (5.34 to 13.67)**	−2.05 (−6.36 to 2.26)
	Treatment	8.85 (7.32 to 10.38)	17.54 (15.14 to 19.93)	23.40 (20.98 to 25.82)	38.43 (35.97 to 40.89)	50.31 (47.06 to 53.56)	**9.55 (8.60 to 10.51)**	**11.88 (7.80 to 15.96)**	2.33 (−1.86 to 6.51)
	Control among prevalence	6.39 (5.04 to 7.73)	12.78 (10.94 to 14.63)	17.94 (15.71 to 20.16)	29.95 (27.72 to 32.18)	41.61 (38.45 to 44.77)	**7.66 (6.82 to 8.50)**	**11.67 (7.80 to 15.53)**	**4.01 (0.05 to 7.97)**
	Control among treatment	51.16 (42.15 to 60.17)	54.30 (47.01 to 61.59)	61.33 (55.66 to 67.01)	66.14 (62.18 to 70.10)	74.78 (71.09 to 78.47)	**5.39 (2.57 to 8.21)**	**8.64 (3.24 to 14.04)**	3.25 (−2.84 to 9.34)
Second quartile	Prevalence	42.63 (40.79 to 44.46)	42.66 (40.80 to 44.53)	45.65 (43.56 to 47.73)	47.58 (45.97 to 49.20)	51.49 (49.43 to 53.54)	**1.81 (1.03 to 2.59)**	**3.90 (1.28 to 6.53)**	2.09 (−0.65 to 4.83)
	Awareness	15.85 (13.92 to 17.78)	24.29 (21.95 to 26.63)	33.16 (30.61 to 35.72)	41.08 (38.77 to 43.38)	49.91 (47.11 to 52.72)	**8.45 (7.48 to 9.42)**	**8.84 (5.20 to 12.47)**	0.39 (−3.37 to 4.15)
	Treatment	6.95 (5.66 to 8.24)	13.88 (12.04 to 15.72)	19.42 (17.32 to 21.52)	28.51 (26.40 to 30.62)	40.91 (38.08 to 43.75)	**7.06 (6.26 to 7.87)**	**12.40 (8.87 to 15.93)**	**5.34 (1.72 to 8.96)**
	Control among prevalence	5.84 (4.63 to 7.04)	10.86 (9.14 to 12.57)	17.81 (15.81 to 19.81)	23.87 (21.78 to 25.96)	33.21 (30.54 to 35.87)	**6.12 (5.35 to 6.90)**	**9.34 (5.95 to 12.72)**	3.22 (−0.26 to 6.69)
	Control among treatment	51.93 (42.75 to 61.11)	52.50 (44.79 to 60.20)	65.96 (60.36 to 71.56)	72.78 (69.07 to 76.50)	74.74 (70.96 to 78.52)	**8.36 (5.52 to 11.19)**	1.95 (−3.34 to 7.25)	**−6.40 (−12.41 to −0.40)**
Third quartile	Prevalence	37.96 (36.19 to 39.72)	40.32 (38.55 to 42.10)	39.95 (37.99 to 41.91)	44.96 (43.31 to 46.60)	45.58 (43.56 to 47.60)	**2.10 (1.33 to 2.87)**	0.62 (−2.00 to 3.24)	−1.48 (−4.20 to 1.25)
	Awareness	15.99 (13.91 to 18.08)	21.63 (19.14 to 24.11)	29.18 (26.56 to 31.80)	36.87 (34.57 to 39.16)	45.98 (43.07 to 48.89)	**7.06 (6.05 to 8.07)**	**9.11 (5.40 to 12.82)**	2.05 (−1.79 to 5.89)
	Treatment	6.13 (4.81 to 7.45)	11.50 (9.69 to 13.31)	16.69 (14.54 to 18.85)	24.31 (22.30 to 26.31)	34.22 (31.36 to 37.08)	**6.03 (5.23 to 6.82)**	**9.91 (6.42 to 13.41)**	**3.89 (0.30 to 7.47)**
	Control among prevalence	5.38 (4.20 to 6.56)	9.10 (7.54 to 10.67)	12.88 (11.08 to 14.69)	21.17 (19.33 to 23.00)	29.66 (27.05 to 32.27)	**5.22 (4.51 to 5.94)**	**8.49 (5.31 to 11.68)**	**3.27 (0.00 to 6.53)**
	Control among treatment	53.16 (41.69 to 64.63)	56.56 (48.66 to 64.47)	59.48 (52.38 to 66.58)	70.68 (66.53 to 74.83)	75.87 (71.96 to 79.77)	**6.54 (3.35 to 9.74)**	5.19 (−0.49 to 10.86)	−1.36 (−7.87 to 5.15)
Highest quartile	Prevalence	39.99 (38.42 to 41.56)	41.28 (39.37 to 43.18)	41.18 (39.35 to 43.02)	43.68 (42.25 to 45.11)	44.72 (42.87 to 46.58)	**1.12 (0.43 to 1.81)**	1.04 (−1.30 to 3.39)	−0.08 (−2.52 to 2.37)
	Awareness	21.85 (19.67 to 24.03)	22.31 (19.81 to 24.81)	28.22 (25.76 to 30.68)	35.29 (33.13 to 37.46)	43.50 (40.69 to 46.31)	**4.72 (3.73 to 5.72)**	**8.21 (4.65 to 11.76)**	3.48 (−0.21 to 7.17)
	Treatment	6.88 (5.50 to 8.26)	11.39 (9.65 to 13.13)	16.80 (14.89 to 18.71)	22.43 (20.56 to 24.30)	32.69 (30.03 to 35.34)	**5.22 (4.47 to 5.98)**	**10.26 (7.01 to 13.51)**	**5.03 (1.70 to 8.37)**
	Control among prevalence	8.20 (6.72 to 9.68)	10.15 (8.53 to 11.76)	14.48 (12.51 to 16.46)	18.90 (17.07 to 20.73)	27.26 (24.55 to 29.96)	**3.68 (2.92 to 4.44)**	**8.36 (5.09 to 11.63)**	**4.68 (1.32 to 8.04)**
	Control among treatment	53.89 (43.57 to 64.21)	56.00 (48.27 to 63.74)	65.35 (59.03 to 71.66)	67.30 (62.86 to 71.74)	72.94 (68.66 to 77.21)	**4.85 (1.72 to 7.98)**	5.63 (−0.55 to 11.82)	0.79 (−6.14 to 7.72)
Smoking status									
Smoker	Prevalence	49.21 (47.81 to 50.61)	48.13 (46.63 to 49.62)	50.40 (48.78 to 52.01)	51.74 (50.49 to 53.00)	53.50 (51.85 to 55.15)	**1.01 (0.41 to 1.61)**	1.76 (−0.31 to 3.83)	0.75 (−1.41 to 2.91)
	Awareness	14.38 (12.89 to 15.87)	19.80 (18.03 to 21.58)	23.61 (21.84 to 25.39)	34.34 (32.70 to 35.98)	42.53 (40.48 to 44.59)	**6.46 (5.74 to 7.17)**	**8.19 (5.57 to 10.82)**	1.74 (−0.98 to 4.46)
	Treatment	4.93 (4.12 to 5.75)	10.67 (9.42 to 11.93)	13.93 (12.50 to 15.36)	22.82 (21.35 to 24.30)	32.21 (30.19 to 34.22)	**5.75 (5.20 to 6.30)**	**9.38 (6.89 to 11.88)**	**3.64 (1.08 to 6.19)**
	Control among prevalence	4.19 (3.42 to 4.97)	7.30 (6.28 to 8.32)	9.23 (8.02 to 10.43)	16.90 (15.63 to 18.16)	23.61 (21.82 to 25.39)	**4.07 (3.60 to 4.55)**	**6.71 (4.52 to 8.90)**	**2.64 (0.40 to 4.88)**
	Control among treatment	39.49 (31.28 to 47.70)	43.87 (37.52 to 50.23)	50.42 (44.28 to 56.56)	61.31 (57.79 to 64.82)	64.96 (61.40 to 68.52)	**8.00 (5.48 to 10.51)**	3.66 (−1.36 to 8.67)	−4.34 (−9.95 to 1.27)
Non-smoker	Prevalence	38.78 (37.56 to 39.99)	38.71 (37.42 to 40.00)	40.11 (38.86 to 41.35)	43.23 (42.10 to 44.36)	44.36 (43.04 to 45.68)	**1.53 (1.00 to 2.06)**	1.13 (−0.63 to 2.89)	−0.40 (−2.24 to 1.43)
	Awareness	20.98 (19.40 to 22.56)	27.53 (25.81 to 29.26)	39.02 (37.05 to 40.98)	46.13 (44.54 to 47.72)	55.03 (52.95 to 57.12)	**8.69 (7.97 to 9.41)**	**8.90 (6.27 to 11.53)**	0.21 (−2.52 to 2.94)
	Treatment	9.25 (8.15 to 10.36)	16.35 (14.84 to 17.87)	23.60 (21.98 to 25.22)	31.99 (30.54 to 33.45)	43.93 (41.90 to 45.96)	**7.58 (6.98 to 8.18)**	**11.94 (9.44 to 14.44)**	**4.36 (1.79 to 6.93)**
	Control among prevalence	9.11 (7.99 to 10.23)	14.27 (12.95 to 15.59)	21.85 (20.26 to 23.44)	28.50 (27.00 to 29.99)	39.79 (37.75 to 41.83)	**6.61 (6.00 to 7.21)**	**11.29 (8.76 to 13.82)**	**4.69 (2.09 to 7.28)**
	Control among treatment	60.27 (54.47 to 66.07)	62.43 (58.01 to 66.84)	70.42 (67.13 to 73.70)	74.50 (72.08 to 76.92)	81.34 (79.12 to 83.57)	**5.24 (3.50 to 6.98)**	**6.85 (3.56 to 10.13)**	1.61 (−2.11 to 5.33)
Waist-to-height ratio^c^									
Normal	Prevalence	27.87 (26.77 to 28.98)	30.08 (28.73 to 31.43)	31.15 (29.84 to 32.46)	31.29 (30.17 to 32.41)	32.02 (30.43 to 33.60)	**1.13 (0.63 to 1.64)**	0.73 (−1.21 to 2.67)	−0.40 (−2.41 to 1.60)
	Awareness	14.87 (13.03 to 16.71)	17.81 (16.04 to 19.58)	25.38 (23.28 to 27.48)	32.20 (30.00 to 34.39)	43.27 (40.58 to 45.96)	**5.99 (5.08 to 6.89)**	**11.07 (7.60 to 14.54)**	**5.08 (1.50 to 8.67)**
	Treatment	4.19 (3.36 to 5.03)	8.82 (7.48 to 10.15)	13.61 (11.94 to 15.27)	18.94 (17.20 to 20.67)	31.21 (28.62 to 33.80)	**7.42 (6.88 to 7.95)**	**12.27 (9.16 to 15.39)**	**7.37 (4.19 to 10.54)**
	Control among prevalence	5.48 (4.32 to 6.65)	9.67 (8.33 to 11.02)	14.75 (13.01 to 16.49)	20.32 (18.62 to 22.03)	30.42 (27.83 to 33.01)	**4.97 (4.31 to 5.63)**	**10.10 (6.99 to 13.20)**	**5.13 (1.95 to 8.30)**
	Control among treatment	59.28 (48.80 to 69.76)	65.95 (58.03 to 73.87)	71.14 (65.14 to 77.14)	78.05 (73.89 to 82.21)	84.12 (80.67 to 87.57)	**6.18 (3.06 to 9.30)**	**6.07 (0.64 to 11.50)**	−0.11 (−6.37 to 6.15)
Central adiposity	Prevalence	55.22 (53.92 to 56.53)	55.48 (54.18 to 56.77)	57.55 (56.23 to 58.87)	59.68 (58.64 to 60.72)	59.93 (58.74 to 61.12)	**1.58 (1.05 to 2.11)**	0.25 (−1.34 to 1.84)	−1.32 (−3.00 to 0.35)
	Awareness	19.44 (18.04 to 20.84)	26.38 (24.77 to 27.99)	34.58 (32.91 to 36.24)	43.84 (42.48 to 45.20)	51.02 (49.24 to 52.80)	**8.19 (7.57 to 8.82)**	**7.18 (4.94 to 9.42)**	−1.02 (−3.34 to 1.31)
	Treatment	8.61 (7.68 to 9.55)	15.71 (14.36 to 17.06)	21.56 (20.13 to 23.00)	31.15 (29.83 to 32.47)	40.80 (39.04 to 42.56)	**8.26 (7.58 to 8.94)**	**9.65 (7.45 to 11.86)**	2.24 (−0.03 to 4.51)
	Control among prevalence	7.01 (6.16 to 7.87)	11.09 (10.06 to 12.12)	16.08 (14.83 to 17.33)	23.91 (22.69 to 25.13)	32.35 (30.70 to 34.00)	**5.66 (5.18 to 6.14)**	**8.44 (6.38 to 10.49)**	**2.78 (0.67 to 4.89)**
	Control among treatment	51.07 (45.51 to 56.62)	51.45 (47.14 to 55.76)	60.24 (56.74 to 63.73)	66.87 (64.56 to 69.17)	71.82 (69.50 to 74.13)	**6.28 (4.60 to 7.95)**	**4.95 (1.68 to 8.22)**	−1.32 (−5.00 to 2.35)
Daily calorie intake^d^									
Low	Prevalence	40.42 (38.96 to 41.89)	41.31 (39.77 to 42.85)	45.45 (43.92 to 46.99)	46.21 (44.87 to 47.54)	47.84 (46.11 to 49.56)	**2.15 (1.52 to 2.78)**	1.63 (−0.57 to 3.83)	−0.52 (−2.81 to 1.77)
	Awareness	18.91 (17.17 to 20.66)	25.80 (23.83 to 27.78)	37.20 (35.08 to 39.33)	46.79 (45.02 to 48.55)	54.77 (52.54 to 56.99)	**9.56 (8.76 to 10.36)**	**7.98 (5.13 to 10.83)**	−1.58 (−4.55 to 1.38)
	Treatment	8.55 (7.36 to 9.74)	16.17 (14.56 to 17.78)	24.25 (22.39 to 26.10)	33.28 (31.59 to 34.97)	44.21 (42.05 to 46.37)	**5.40 (4.84 to 5.96)**	**10.93 (8.18 to 13.68)**	2.67 (−0.16 to 5.50)
	Control among prevalence	7.77 (6.63 to 8.91)	12.82 (11.41 to 14.22)	19.38 (17.75 to 21.00)	27.39 (25.79 to 29.00)	38.04 (35.84 to 40.25)	**6.61 (5.97 to 7.25)**	**10.65 (7.91 to 13.38)**	**4.04 (1.23 to 6.85)**
	Control among treatment	55.64 (48.28 to 63.00)	58.81 (53.63 to 63.99)	64.43 (60.43 to 68.44)	69.67 (66.69 to 72.66)	78.93 (76.37 to 81.49)	**5.02 (2.87 to 7.16)**	**9.25 (5.33 to 13.18)**	4.24 (−0.24 to 8.71)
High	Prevalence	39.99 (38.72 to 41.26)	38.41 (37.10 to 39.72)	38.47 (37.21 to 39.73)	41.03 (39.91 to 42.15)	41.17 (39.69 to 42.65)	0.37 (−0.17 to 0.91)	0.14 (−1.73 to 2.01)	−0.23 (−2.18 to 1.72)
	Awareness	17.41 (15.83 to 18.99)	22.58 (20.72 to 24.44)	27.78 (25.92 to 29.65)	35.66 (33.99 to 37.32)	45.36 (43.04 to 47.68)	**6.04 (5.30 to 6.78)**	**9.70 (6.85 to 12.56)**	**3.66 (0.71 to 6.61)**
	Treatment	6.55 (5.60 to 7.50)	12.22 (10.82 to 13.63)	15.14 (13.73 to 16.55)	23.40 (21.97 to 24.82)	34.02 (31.72 to 36.32)	**6.99 (6.53 to 7.46)**	**10.63 (7.92 to 13.34)**	**5.23 (2.46 to 8.00)**
	Control among prevalence	6.13 (5.18 to 7.08)	9.62 (8.48 to 10.76)	13.34 (11.99 to 14.69)	19.85 (18.52 to 21.17)	28.76 (26.61 to 30.91)	**4.53 (4.01 to 5.06)**	**8.91 (6.38 to 11.44)**	**4.38 (1.80 to 6.96)**
	Control among treatment	51.13 (43.92 to 58.34)	51.12 (45.09 to 57.14)	60.59 (55.53 to 65.65)	69.00 (65.66 to 72.33)	72.87 (69.66 to 76.07)	**7.15 (4.84 to 9.46)**	3.87 (−0.76 to 8.50)	−3.28 (−8.46 to 1.89)
High-risk drinking^e^									
No	Prevalence	43.37 (42.39 to 44.35)	42.90 (41.85 to 43.94)	43.30 (42.19 to 44.40)	46.63 (45.71 to 47.54)	47.69 (46.59 to 48.79)	**1.08 (0.65 to 1.50)**	1.06 (−0.38 to 2.51)	−0.01 (−1.52 to 1.49)
	Awareness	17.42 (16.24 to 18.60)	24.13 (22.71 to 25.56)	32.92 (31.45 to 34.39)	41.59 (40.32 to 42.86)	50.93 (49.27 to 52.59)	**8.16 (7.60 to 8.72)**	**9.34 (7.25 to 11.44)**	1.18 (−0.99 to 3.35)
	Treatment	7.43 (6.64 to 8.21)	14.23 (13.08 to 15.38)	20.00 (18.78 to 21.22)	28.67 (27.50 to 29.85)	40.01 (38.32 to 41.70)	**5.12 (4.10 to 6.15)**	**11.34 (9.28 to 13.40)**	**4.34 (2.23 to 6.45)**
	Control among prevalence	6.67 (5.92 to 7.42)	11.42 (10.50 to 12.35)	16.82 (15.68 to 17.97)	23.87 (22.77 to 24.97)	33.88 (32.25 to 35.51)	**5.74 (5.32 to 6.17)**	**10.01 (8.04 to 11.98)**	**4.27 (2.25 to 6.28)**
	Control among treatment	52.96 (47.93 to 57.99)	56.66 (52.72 to 60.60)	64.24 (61.19 to 67.30)	70.17 (68.06 to 72.27)	75.99 (73.97 to 78.01)	**6.16 (4.63 to 7.68)**	**5.82 (2.91 to 8.74)**	−0.33 (−3.63 to 2.96)
Yes	Prevalence	45.40 (42.81 to 47.99)	45.10 (42.23 to 47.96)	52.82 (49.76 to 55.88)	49.35 (46.94 to 51.75)	52.99 (49.96 to 56.02)	**1.90 (0.76 to 3.03)**	3.65 (−0.21 to 7.51)	1.75 (−2.27 to 5.78)
	Awareness	17.54 (14.21 to 20.87)	18.80 (15.52 to 22.09)	21.78 (18.49 to 25.07)	32.59 (29.46 to 35.72)	36.44 (32.85 to 40.02)	**4.96 (3.51 to 6.41)**	3.84 (−0.93 to 8.62)	−1.12 (−6.10 to 3.87)
	Treatment	4.22 (2.66 to 5.79)	8.42 (6.24 to 10.60)	11.29 (8.75 to 13.82)	20.10 (17.37 to 22.84)	26.84 (23.61 to 30.07)	**4.97 (4.31 to 5.63)**	**6.73 (2.49 to 10.98)**	1.61 (−2.76 to 5.98)
	Control among prevalence	5.23 (3.44 to 7.03)	6.00 (4.16 to 7.85)	8.66 (6.46 to 10.85)	16.25 (13.87 to 18.63)	19.62 (16.69 to 22.56)	**3.68 (2.72 to 4.63)**	3.37 (−0.42 to 7.16)	−0.30 (−4.21 to 3.60)
	Control among treatment	49.13 (28.87 to 69.40)	34.78 (22.12 to 47.44)	53.38 (40.78 to 65.99)	60.56 (52.86 to 68.25)	62.41 (55.35 to 69.47)	**7.93 (2.19 to 13.67)**	1.86 (−8.57 to 12.28)	−6.07 (−17.97 to 5.83)

BMI, body mass index; CI confidence interval; KNHANES, Korea National Health and Nutrition Examination Survey.

Numbers in bold indicate a significant difference (*P* < 0.05).

This table presents the percentages of prevalence, awareness, treatment, control among dyslipidemia, and control among treatment for the overall population and each subgroup, along with trends (β) before and during the pandemic, as well as differences in trends (β_diff_).

^a^All βs and β_diffs_ were expressed by multiplying 100.

^b^BMI was divided into four groups according to Asian-Pacific guidelines: underweight (< 18.5 kg/m^2^), normal (18.5–22.9 kg/m^2^), overweight (23.0–24.9 kg/m^2^), and obese (≥ 25 kg/m^2^).

^c^Waist-to-height ratio was calculated as waist circumference divided by height and categorized into two groups: normal (< 0.5) and central adiposity (≥ 0.5).

^d^Daily calorie intake was categorized into two groups: low (below the median) and high (above the median).

^e^High-risk drinking was defined as consuming ≥ 7 drinks per occasion for men or ≥ 5 for women at least twice per week, classified as ‘yes’ or ‘no’ accordingly.

**Table 3:** Weighted odds ratios of prevalence, awareness, treatment, control among dyslipidemia, and control among treatment in each socioeconomic factor based on data obtained from the KNHANES.

Times	Prevalence		Awareness		Treatment		Control among prevalence		Control among treatment	
	Weighted OR (95% CI)	*P*-value	Weighted OR (95% CI)	*P*-value	Weighted OR (95% CI)	*P*-value	Weighted OR (95% CI)	*P*-value	Weighted OR (95% CI)	*P*-value
Sex										
Female	1.00 (ref)		1.00 (ref)		1.00 (ref)		1.00 (ref)		1.00 (ref)	
Male	**1.59 (1.54 to 1.65)**	**< 0.001**	**0.51 (0.48 to 0.54)**	**< 0.001**	**0.51 (0.48 to 0.54)**	**< 0.001**	**0.40 (0.38 to 0.43)**	**< 0.001**	**0.48 (0.43 to 0.54)**	**< 0.001**
Age, years										
30–39	1.00 (ref)		1.00 (ref)		1.00 (ref)		1.00 (ref)		1.00 (ref)	
40–49	**1.46 (1.38 to 1.54)**	**< 0.001**	**2.62 (2.29 to 2.99)**	**< 0.001**	**3.69 (2.98 to 4.56)**	**< 0.001**	**2.78 (2.27 to 3.41)**	**< 0.001**	**1.68 (1.09 to 2.59)**	**0.018**
50–59	**2.39 (2.27 to 2.52)**	**< 0.001**	**5.84 (5.15 to 6.63)**	**< 0.001**	**9.53 (7.80 to 11.64)**	**< 0.001**	**6.23 (5.16 to 7.52)**	**< 0.001**	**1.90 (1.27 to 2.85)**	**0.002**
60–69	**3.25 (3.07 to 3.44)**	**< 0.001**	**10.00 (8.80 to 11.36)**	**< 0.001**	**18.62 (15.28 to 22.68)**	**< 0.001**	**11.92 (9.89 to 14.37)**	**< 0.001**	**2.40 (1.62 to 3.56)**	**< 0.001**
≥ 70	**2.96 (2.81 to 3.13)**	**< 0.001**	**8.35 (7.34 to 9.50)**	**< 0.001**	**18.36 (15.03 to 22.44)**	**< 0.001**	**11.11 (9.22 to 13.40)**	**< 0.001**	**2.62 (1.76 to 3.89)**	**< 0.001**
Region of residence										
Urban	1.00 (ref)		1.00 (ref)		1.00 (ref)		1.00 (ref)		1.00 (ref)	
Rural	**1.13 (1.08 to 1.19)**	**< 0.001**	**0.87 (0.81 to 0.95)**	**0.001**	**0.89 (0.81 to 0.98)**	**0.018**	**0.85 (0.77 to 0.94)**	**0.002**	**0.85 (0.74 to 0.98)**	**0.026**
BMI group^a^										
Underweight	1.00 (ref)		1.00 (ref)		1.00 (ref)		1.00 (ref)		1.00 (ref)	
Normal	**1.92 (1.71 to 2.15)**	**< 0.001**	**1.37 (1.09 to 1.73)**	**0.007**	**1.41 (1.07 to 1.85)**	**0.015**	1.08 (0.83 to 1.41)	0.551	0.90 (0.49 to 1.66)	0.746
Overweight	**3.55 (3.16 to 3.98)**	**< 0.001**	**1.47 (1.16 to 1.85)**	**0.001**	**1.53 (1.16 to 2.02)**	**0.003**	0.99 (0.76 to 1.29)	0.951	0.63 (0.34 to 1.15)	0.133
Obese	**5.62 (5.01 to 6.30)**	**< 0.001**	**1.49 (1.18 to 1.88)**	**0.001**	**1.62 (1.23 to 2.14)**	**0.001**	0.87 (0.67 to 1.14)	0.312	**0.44 (0.24 to 0.81)**	**0.008**
Educational background										
College or higher	1.00 (ref)		1.00 (ref)		1.00 (ref)		1.00 (ref)		1.00 (ref)	
High school	**1.22 (1.17 to 1.27)**	**< 0.001**	**1.41 (1.32 to 1.51)**	**< 0.001**	**1.49 (1.37 to 1.62)**	**< 0.001**	**1.40 (1.28 to 1.52)**	**< 0.001**	1.01 (0.86 to 1.18)	0.914
Middle school	**1.72 (1.63 to 1.82)**	**< 0.001**	**2.18 (2.01 to 2.37)**	**< 0.001**	**2.32 (2.12 to 2.55)**	**< 0.001**	**2.11 (1.91 to 2.33)**	**< 0.001**	1.02 (0.86 to 1.22)	0.829
Elementary school or lower	**1.95 (1.87 to 2.04)**	**< 0.001**	**1.98 (1.84 to 2.13)**	**< 0.001**	**2.36 (2.16 to 2.57)**	**< 0.001**	**1.96 (1.79 to 2.14)**	**< 0.001**	0.95 (0.82 to 1.11)	0.545
Household income										
Highest quartile	1.00 (ref)		1.00 (ref)		1.00 (ref)		1.00 (ref)		1.00 (ref)	
Third quartile	0.99 (0.95 to 1.04)	0.646	1.00 (0.93 to 1.08)	0.931	1.04 (0.95 to 1.14)	0.384	1.01 (0.92 to 1.12)	0.770	1.07 (0.91 to 1.27)	0.393
Second quartile	**1.16 (1.11 to 1.22)**	**< 0.001**	**1.13 (1.05 to 1.21)**	**0.002**	**1.25 (1.14 to 1.36)**	**< 0.001**	**1.17 (1.07 to 1.29)**	**0.001**	1.09 (0.93 to 1.27)	0.305
Lowest quartile	**1.60 (1.52 to 1.68)**	**< 0.001**	**1.47 (1.36 to 1.59)**	**< 0.001**	**1.69 (1.55 to 1.85)**	**< 0.001**	**1.45 (1.32 to 1.59)**	**< 0.001**	0.95 (0.81 to 1.11)	0.494
Smoking status										
Non-smoker	1.00 (ref)		1.00 (ref)		1.00 (ref)		1.00 (ref)		1.00 (ref)	
Smoker	**1.46 (1.41 to 1.51)**	**< 0.001**	**0.59 (0.56 to 0.62)**	**< 0.001**	**0.59 (0.56 to 0.63)**	**< 0.001**	**0.47 (0.44 to 0.50)**	**< 0.001**	**0.48 (0.43 to 0.54)**	**< 0.001**
Waist-to-height ratio^b^										
Normal	1.00 (ref)		1.00 (ref)		1.00 (ref)		1.00 (ref)		1.00 (ref)	
Central adiposity	**3.14 (3.03 to 3.25)**	**< 0.001**	**1.59 (1.50 to 1.68)**	**< 0.001**	**0.67 (0.61 to 0.72)**	**< 0.001**	**1.24 (1.16 to 1.33)**	**< 0.001**	**0.56 (0.49 to 0.65)**	**< 0.001**
Daily calorie intake^c^										
Low	1.00 (ref)		1.00 (ref)		1.00 (ref)		1.00 (ref)		1.00 (ref)	
High	0.98 (0.95 to 1.02)	0.286	**0.70 (0.67 to 0.74)**	**< 0.001**	**0.64 (0.60 to 0.68)**	**< 0.001**	**0.67 (0.63 to 0.72)**	**< 0.001**	**0.84 (0.74 to 0.94)**	**0.003**
High-risk drinking^d^										
No	1.00 (ref)		1.00 (ref)		1.00 (ref)		1.00 (ref)		1.00 (ref)	
Yes	**1.18 (1.12 to 1.25)**	**< 0.001**	**0.67 (0.61 to 0.72)**	**< 0.001**	**0.59 (0.53 to 0.65)**	**< 0.001**	**0.55 (0.50 to 0.62)**	**< 0.001**	**0.60 (0.50 to 0.73)**	**< 0.001**

BMI, body mass index; CI confidence interval; KNHANES, Korea National Health and Nutrition Examination Survey; OR, odds ratio.

Numbers in bold indicate a significant difference (*P* < 0.05).

This table presents the weighted odds ratios for the prevalence, awareness, treatment, control among dyslipidemia, and control among treatment, relative to the reference group.

^a^BMI was divided into four groups according to Asian-Pacific guidelines: underweight (< 18.5 kg/m^2^), normal (18.5–22.9 kg/m^2^), overweight (23.0–24.9 kg/m^2^), and obese (≥ 25 kg/m^2^).

^b^Waist-to-height ratio was calculated as waist circumference divided by height and categorized into two groups: normal (< 0.5) and central adiposity (≥ 0.5).

^c^Daily calorie intake was categorized into two groups: low (below the median) and high (above the median).

^d^High-risk drinking was defined as consuming ≥ 7 drinks per occasion for men or ≥ 5 for women at least twice per week, classified as ‘yes’ or ‘no’ accordingly.

### Awareness, treatment, and control of dyslipidemia

Table 2 also shows the trends and differences in awareness, treatment, and control of dyslipidemia. The awareness increased from 17.87% (95% CI 16.75–18.99) to 48.90% (95% CI 47.34–50.47). The treatment began at 7.10% (95% CI 6.39–7.80) and showed a substantial increase over the past 18 years, reaching 38.19% (95% CI 36.61–39.76), with a particularly notable rise during the COVID-19 period (β_diff_, 3.94 [95% CI 1.97–5.92]). Likewise, control among prevalence showed a significant increase from 6.49% (95% CI 5.79–7.19) to 31.82% (95% CI 30.33–33.32), with further enhancing of this trend during the pandemic (β_diff_, 3.52 [95% CI 1.67–5.38]). In addition, control among treatment steadily increased from 52.55% (95% CI 47.61–57.49) to 74.55% (95% CI 72.56–76.55).

Table 3 shows the ORs for the awareness, treatment, and control of dyslipidemia across various socioeconomic groups using data aggregated from 2005 to 2022. Males had lower awareness (wOR, 0.51 [95% CI 0.48–0.54]), treatment (wOR, 0.51 [95% CI 0.48–0.54]), control among prevalence (wOR, 0.40 [95% CI 0.38–0.43]), and control among treatment (wOR, 0.48 [95% CI 0.43–0.54]). Similarly, smokers had lower awareness (wOR, 0.59 [95% CI 0.56–0.62]), treatment (wOR, 0.59 [95% CI 0.56–0.63]), control among prevalence (wOR, 0.47 [95% CI 0.44–0.50]), and control among treatment (wOR, 0.48 [95% CI 0.43–0.54]). Likewise, high-risk drinkers had lower awareness (wOR, 0.67 [95% CI 0.61–0.72]), treatment (wOR, 0.59 [95% CI 0.53–0.65]), control among prevalence (wOR, 0.55 [95% CI 0.50–0.62]), and control among treatment (wOR, 0.60 [95% CI 0.50–0.73]). Compared to the younger population, the older population had significantly higher awareness (60–69 years: wOR, 10.00 [95% CI 8.80–11.36] and ≥ 70 years: wOR, 8.35 [95% CI 7.34–9.50]), treatment (60–69 years: wOR, 18.62 [95% CI 15.28–22.68] and ≥ 70 years: wOR, 18.36 [95% CI 15.03–22.44]), and control among prevalence (60–69 years: wOR, 11.92 [95% CI 9.89–14.37] and ≥ 70 years: wOR, 11.11 [95% CI 9.22–13.40]). The underweight population had lower awareness and treatment compared to other populations. Overall, awareness, treatment, and control among prevalence showed similar patterns.

Tables S4 to S7 present the ORs and ratio of ORs (RORs) for each socioeconomic subgroup before and during the COVID-19 pandemic. During the pandemic, awareness significantly increased among older adults subgroups (ROR, 3.15 [95% CI 2.27–4.38]), individuals with lower educational background (ROR, 1.81 [95% CI 1.50–2.18]), and those with lower household income (ROR, 1.48 [95% CI 1.22–1.80]). Additionally, control among the total prevalence also showed a significant increase in these subgroups (≥ 70 years: ROR, 1.94 [95% CI 1.24–3.04], elementary school or lower: ROR, 1.49 [95% CI 1.22–1.82], and lowest household income: wOR, 1.36 [95% CI 1.10–1.66]).

## Discussion

### Key finding

In this study, we examined a comprehensive longitudinal trend analysis of the prevalence, awareness, treatment, and control of dyslipidemia among a total of 98,396 Korean adults. We observed a consistent increase in dyslipidemia from 2005 to 2022. Awareness and control among treatment continued to show a significant increasing trend, in line with the prevalence of dyslipidemia. Over the past 18 years, the treatment and control rates among individuals with dyslipidemia showed a substantial increasing trend. Notably, during the COVID-19 pandemic, this trend appeared to intensify, with a steeper rise observed in the pandemic period compared to the pre-pandemic period. The prevalence of dyslipidemia was higher among males, older populations, rural residents, those with a higher BMI, lower educational attainment, lower household income, smokers, individuals with central adiposity, and those engaging in high-risk alcohol consumption. Lower awareness, treatment, and control rates among individuals with dyslipidemia were associated with male sex, younger population, rural residency, higher educational attainment, higher household income, smoking, and high-risk alcohol consumption. This study analyzed trends in dyslipidemia, identified the association between the COVID-19 pandemic and high-risk groups, and suggested specific management strategies at the national level.

### Comparison with previous studies

Many previous studies have examined the prevalence of dyslipidemia in different countries and regions. Jordan (81.6%; *n* = 3132) and Ethiopia (66.7%; *n* = 321) had a higher prevalence than South Korea, while China (31.2%; *n* = 65,128) had a lower prevalence than South Korea^22–24^. This Chinese study identified common risk factors for dyslipidemia, such as male sex, obesity, and smoking, in line with our research. However, contrary to our findings, a higher prevalence was noted among urban residents^24^. As of research in China (*n* = 135,403), the awareness of dyslipidemia was higher than ours (64%; *n* = 36,958), with similar treatment (18.9%; *n* = 6993) but lower control among prevalence (7.2%; *n* = 504)^25^. Additionally, awareness, treatment, and control among prevalence were higher among females than males, consistent with our findings^25^.

The substantial differences in prevalence between countries are likely due to variations in ethnicity, dietary habits, lifestyle, and access to healthcare^24,26^. Previous studies have mainly examined the prevalence and risk factors of dyslipidemia in each region, and some additionally assessed awareness, treatment, and control^25,27^. However, most studies were generally based on data from a specific year^27,28^. In contrast, our research has investigated changes over the past 18 years in a large population and analyzed the influence of the COVID-19 pandemic on these trends. We also examined the trends among groups disproportionately affected by the pandemic. Findings from this long-term study should contribute to developing public health policies to manage dyslipidemia across various socioeconomic groups.

South Korea’s trends in dyslipidemia may differ from other nations due to a combination of healthcare system characteristics, lifestyle factors, and genetic predispositions. The country’s universal healthcare system ensures broad access to medical services, including lipid-lowering treatments, which may contribute to higher treatment and control rates compared to nations with less accessible healthcare systems. Additionally, dietary patterns in South Korea have shifted significantly in recent decades, with an increase in processed and high-calorie food consumption, which may uniquely influence dyslipidemia prevalence^29,30^. Lastly, genetic predispositions specific to East Asian populations, such as differences in lipid metabolism, may affect both prevalence and treatment response^31^. These factors highlight the need for country-specific public health strategies in managing dyslipidemia.

### Plausible underlying mechanisms

In South Korea, the consumption of ultra-processed foods has been increasing^30^, and physical activity declined during the COVID-19 pandemic^32^. These factors may have contributed to the rising prevalence of dyslipidemia. Awareness would have increased as public awareness about dyslipidemia and healthcare utilization increased^33–35^. Control among treatments could have increased due to the development of anti-dyslipidemia medications^36–38^.

The treatment and control rates of dyslipidemia have steadily increased, with this trend accelerating during the COVID-19 pandemic. In South Korea, the decline in healthcare utilization due to COVID-19 was relatively lower than the global average^34^. Instead, heightened awareness about dyslipidemia and health concerns during the pandemic may have contributed to this rapid increase^39^. Additionally, the expansion of telemedicine services, digital health interventions, and increased accessibility to medication refills may have played a role in improving treatment adherence and disease management during this period^34,35^. Control among prevalence would have risen as people recognized the need to manage dyslipidemia and began receiving medication treatment. Indeed, control among prevalence has shown a similar trend to the treatment. These indicators are closely related to medication treatment, which can be initiated immediately; therefore, as people became more health-conscious during the pandemic, these measures likely increased immediately.

The risk factors for dyslipidemia included male sex, older population, rural residence, higher BMI, lower education and income levels, and smoking. Male sex was also a risk factor in most other studies. Males may have a higher prevalence because they are more likely to be obese and smoke^11,40^. Eight sex-biased genes are genetically associated with polygenic dyslipidemia, which puts males at higher risk of dyslipidemia^41^. In the elderly population, TC and LDL-C levels increase with age, which may increase prevalence^42^. Lower education and income levels are associated with a higher prevalence of chronic diseases and lower health awareness, which may lead to a higher prevalence of dyslipidemia^43^. High BMI and smoking are well-known risk factors for dyslipidemia^24^.

Low awareness, treatment, and control among prevalence are associated with male sex, younger population, rural residence, higher education and income levels, and smoking. These three indicators show similar trends. When diagnosed with dyslipidemia, individuals are more likely to receive treatment, and if treated, they are more likely to achieve control^44^. Males may have lower awareness due to less frequent healthcare utilization^34^. Younger populations, often perceiving themselves as low-risk, may not seek screening or treatment, resulting in lower management rates^45^. Rural residents may face limited access to healthcare services, contributing to lower awareness, treatment, and control rates^46^.

Interestingly, individuals with higher education and income levels also showed lower awareness and treatment rates. One possible explanation is that they may perceive themselves as being at lower risk due to healthier lifestyles or overall well-being. Additionally, demanding work schedules and different healthcare-seeking behaviors might lead to fewer routine check-ups. These findings suggest the need for targeted public health strategies to encourage regular screenings and risk awareness among this demographic^43,47^. Obesity is a well-known risk factor for dyslipidemia, so individuals with higher BMI tend to have greater awareness and treatment rates. However, weight loss is often recommended for obese individuals to manage dyslipidemia, meaning that despite higher treatment rates, control rates may not significantly improve^48^.

During the COVID-19 pandemic, the treatment and control rates of dyslipidemia increased at an accelerated pace. Several potential mechanisms may explain this trend. First, the heightened public awareness of health risks during the pandemic likely led to more proactive management of chronic conditions, including dyslipidemia. Increased concerns over metabolic health, particularly given the association between COVID-19 severity and cardiometabolic disorders, may have driven more individuals to seek medical consultations and adhere to lipid-lowering treatments^6^. Second, healthcare system adaptations, such as expanded telemedicine services and more flexible prescription regulations, may have improved accessibility to dyslipidemia treatment, facilitating better adherence and follow-up care^34^. These factors collectively contributed to the observed trend of improved dyslipidemia management during the pandemic.

### Strengths and limitations

This study has several limitations due to the inherent characteristics of the KNHANES. First, each variable used in our research is not independent. Because several variables can comprehensively affect dyslipidemia, it is difficult to conclude that one variable directly affects dyslipidemia^49^. Second, as the KNHANES dataset is derived from a survey, it is inherently subject to individual bias, and the results may be influenced by participants’ recall. To mitigate these limitations, we endeavored to incorporate variables defined by objective measurements, such as blood tests, whenever feasible. Third, when evaluating lipid-lowering medication use, standard research practices often assess adherence based on the past two weeks. However, the KNHANES dataset we used does not specify a two-week period in its questionnaire. Instead, it categorizes medication use into broader timeframes, which may introduce slight inconsistencies in adherence assessment. To address this limitation, we focused on overall trends rather than short-term adherence, allowing for a broader understanding of dyslipidemia management patterns in the population. Finally, since this study only used data from South Korea, there may be limitations in applying our findings to other ethnicities or countries^2^. Therefore, similar follow-up studies using data from different countries are needed. However, this study has significant strengths. We provide a comprehensive insight into dyslipidemia by examining the prevalence of dyslipidemia with its risk factors over the past 18 years using a large sample in South Korea, as well as awareness, treatment, and control. We also considered the association of COVID-19 with dyslipidemia by observing changes in each trend between the pre-pandemic and the pandemic, suggesting post-pandemic public health policy development.

### Clinical policy implications

According to our research, positive indicators such as awareness, treatment, and control of dyslipidemia in South Korea have increased, showing that dyslipidemia is being managed more effectively than before. However, the prevalence continues to rise, necessitating consistent management. To further improve dyslipidemia prevention and treatment outcomes, public health policies should focus on expanding screening programs, particularly for younger adults and high-risk groups such as males, smokers, and individuals with obesity. Additionally, targeted health campaigns and education programs should be developed to increase awareness and encourage proactive management of dyslipidemia. Improving healthcare accessibility in rural areas through telemedicine services and community-based interventions could also help address the lower awareness and treatment rates observed in these populations. Additionally, utilizing predictive modeling to identify populations vulnerable to dyslipidemia and facilitate their management could be beneficial^50–52^. These strategies could strengthen the country’s efforts to reduce the burden of dyslipidemia and its associated cardiovascular risks. The risk factors identified in this study should be used to develop more efficient dyslipidemia management policies. In the case of the older population, there was high awareness, treatment, and control among prevalence, as the risk of dyslipidemia was known to be high. Conversely, the younger population, considered to have a lower risk, has been in a management blind spot, resulting in significantly lower management rates. Therefore, the results of these studies should be utilized to assess the risk levels of each indicator by group and establish policies for effectively managing dyslipidemia^53^.

## Conclusion

Over the past 18 years, the prevalence, awareness, treatment, and control of dyslipidemia in South Korea have significantly increased. During the pandemic, the treatment and control among prevalence showed a faster increasing trend than before. This suggests that the pandemic positively influenced the initiation of pharmacological therapy for dyslipidemia. Since the risk of dyslipidemia varies based on factors such as sex, age, region of residence, BMI, central adiposity, educational background, household income, smoking status, and high-risk alcohol consumption, these factors should be considered to assess individual risks and develop national policies for targeted management of dyslipidemia.

## Electronic supplementary material

Below is the link to the electronic supplementary material.

Supplementary Material 1

## Data Availability

The data are available upon request. Study protocol and statistical code: Available from DKY (yonkkang@gmail.com). Dataset: Available from the Korea Disease Control Agency (KDCA) through a data use agreement.
