# Supplementary material for: National trends in dyslipidemia prevalence, awareness, treatment, and control in South Korea from 2005 to 2022
Source: Sci Rep. 2025 May 9;15:16148. doi: 10.1038/s41598-025-00354-2 (PMC12062479; doi:10.1038/s41598-025-00354-2)
Supplement: Supplementary file 1 — Supplementary Material 1 [file 41598_2025_354_MOESM1_ESM.docx]

| Supplementary Material |
| --- |

Original Article

**National Trends in dyslipidemia prevalence, awareness, treatment, and control in South Korea from 2005 to 2022**

**Running title:** Dyslipidemia in South Korea

Hyeseung Lee^1,2†^, Seokjun Kim^1,2†^, Yejun Son^2,3†^, Soeun Kim^2,3^, Hyeon Jin Kim^2,3^, Hyesu Jo^2,4^, Jaeyu Park^2,3^, Kyeongmin Lee^2,4^, Hayeon Lee^2,5^, Jiseung Kang^6,7^, Selin Woo^1,2^, Sunyoung Kim^8^, Sang Youl Rhee^1,2,3,4,9^, Jiyoung Hwang^1,2*^, Lee Smith^10*^, Dong Keon Yon^1,2,3,4,11*^

^†^ These authors contributed equally to this work as the first author.

* These authors contributed equally to this work as the corresponding author.

Corresponding authors:

**Dong Keon Yon, MD, PhD, FACAAI, FAAAAI, ATSF (lead contact)**

Department of Pediatrics, Kyung Hee University College of Medicine, 23 Kyungheedae–ro, Dongdaemun–gu, Seoul 02447, South Korea

Tel: +82–2–6935–2476

Fax: +82–504–478–0201

Email: yonkkang@gmail.com

**Table S1.** Weighted response rates for total cholesterol, low-density lipoprotein cholesterol, high-density lipoprotein cholesterol, and triglycerides based on data from the KNHANES from 2005 to 2022 (n=98,396).

|  | Total | 2005–2009 | 2010–2012 | 2013–2015 | 2016–2019 | 2020-2022 |
| --- | --- | --- | --- | --- | --- | --- |
| Overall, n (%) | 98,396 | 35,005 | 15,610 | 13,440 | 20,601 | 13,740 |
| Total cholesterol | 94.77 (94.58 to 94.97) | 89.93 (89.44 to 90.42) | 95.25 (94.80 to 95.69) | 92.94 (92.36 to 93.52) | 97.27 (96.97 to 97.58) | 97.63 (97.26 to 97.99) |
| Low-density lipoprotein cholesterol | 94.75 (94.56 to 94.94) | 89.83 (89.34 to 90.32) | 95.25 (94.80 to 95.69) | 92.94 (92.36 to 93.52) | 97.26 (96.95 to 97.56) | 97.63 (97.26 to 97.99) |
| High-density lipoprotein cholesterol | 94.77 (94.57 to 94.96) | 89.93 (89.44 to 90.42) | 95.25 (94.80 to 95.69) | 92.94 (92.36 to 93.52) | 97.25 (96.94 to 97.55) | 97.63 (97.26 to 97.99) |
| Triglycerides | 94.77 (94.58 to 94.97) | 89.93 (89.44 to 90.42) | 95.25 (94.80 to 95.69) | 92.94 (92.36 to 93.52) | 97.27 (96.97 to 97.58) | 97.63 (97.26 to 97.99) |

Abbreviations: KNHANES, Korea National Health and Nutrition Examination Survey.

**Table S2.** Weighted odds ratios of prevalence, awareness, treatment, control among dyslipidemia, and control among treatment across socioeconomic factors with interaction by sex.

| Times | Prevalence | | Awareness | | Treatment | | Control among prevalence | | Control among treatment | |
| --- | --- | --- | --- | --- | --- | --- | --- | --- | --- | --- |
|  | Weighted OR (95% CI) | P-value | Weighted OR (95% CI) | P-value | Weighted OR (95% CI) | P-value | Weighted OR (95% CI) | P-value | Weighted OR (95% CI) | P-value |
| Age, years | | | | | | | | | | |
| 30-39 | 1.00 (ref) |  | 1.00 (ref) |  | 1.00 (ref) |  | 1.00 (ref) |  | 1.00 (ref) |  |
| 40-49 | 0.96 (0.86 to 1.08) | 0.494 | 0.81 (0.61 to 1.06) | 0.129 | 1.04 (0.66 to 1.63) | 0.875 | 0.98 (0.66 to 1.45) | 0.928 | 1.02 (0.41 to 2.53) | 0.969 |
| 50-59 | **0.46 (0.41 to 0.52)** | **<.001** | **0.57 (0.44 to 0.74)** | **<.001** | 0.74 (0.49 to 1.13) | 0.164 | 0.74 (0.50 to 1.08) | 0.122 | 0.79 (0.33 to 1.87) | 0.592 |
| 60-69 | **0.26 (0.23 to 0.29)** | **<.001** | **0.46 (0.35 to 0.60)** | **<.001** | **0.59 (0.39 to 0.90)** | **0.014** | 0.76 (0.52 to 1.10) | 0.150 | 1.01 (0.44 to 2.34) | 0.982 |
| ≥70 | **0.22 (0.19 to 0.25)** | **<.001** | **0.53 (0.41 to 0.69)** | **<.001** | **0.65 (0.43 to 0.98)** | **0.041** | 0.92 (0.63 to 1.34) | 0.654 | 1.36 (0.58 to 3.18) | 0.475 |
| Region of residence | | | | | | | | | | |
| Urban | 1.00 (ref) |  | 1.00 (ref) |  | 1.00 (ref) |  | 1.00 (ref) |  | 1.00 (ref) |  |
| Rural | **0.84 (0.77 to 0.91)** | **<.001** | 1.11 (0.97 to 1.26) | 0.121 | 1.10 (0.95 to 1.27) | 0.212 | 1.06 (0.91 to 1.25) | 0.433 | 0.96 (0.73 to 1.27) | 0.781 |
| BMI group ^a^ | | | | | | | | | | |
| Underweight | 1.00 (ref) |  | 1.00 (ref) |  | 1.00 (ref) |  | 1.00 (ref) |  | 1.00 (ref) |  |
| Normal | 1.12 (0.87 to 1.44) | 0.370 | 0.70 (0.41 to 1.18) | 0.181 | 0.56 (0.30 to 1.07) | 0.080 | 0.97 (0.48 to 1.94) | 0.924 | 1.03 (0.28 to 3.84) | 0.966 |
| Overweight | 0.99 (0.77 to 1.28) | 0.946 | 0.63 (0.37 to 1.08) | 0.091 | **0.44 (0.23 to 0.85)** | **0.014** | 0.78 (0.39 to 1.58) | 0.495 | 1.14 (0.30 to 4.35) | 0.846 |
| Obese | 1.06 (0.83 to 1.36) | 0.644 | 0.62 (0.37 to 1.05) | 0.075 | **0.43 (0.23 to 0.82)** | **0.010** | 0.71 (0.36 to 1.41) | 0.335 | 1.05 (0.28 to 3.90) | 0.939 |
| Educational background | | | | | | | | | | |
| College or higher | 1.00 (ref) |  | 1.00 (ref) |  | 1.00 (ref) |  | 1.00 (ref) |  | 1.00 (ref) |  |
| High school | **0.69 (0.64 to 0.76)** | **<.001** | **0.77 (0.67 to 0.89)** | **<.001** | 0.93 (0.80 to 1.09) | 0.388 | **0.80 (0.68 to 0.95)** | **0.013** | 0.81 (0.57 to 1.13) | 0.218 |
| Middle school | **0.43 (0.39 to 0.48)** | **<.001** | **0.59 (0.50 to 0.70)** | **<.001** | **0.74 (0.61 to 0.89)** | **0.001** | **0.71 (0.59 to 0.87)** | **0.001** | 1.10 (0.77 to 1.58) | 0.600 |
| Elementary school or lower | **0.28 (0.25 to 0.31)** | **<.001** | **0.57 (0.49 to 0.66)** | **<.001** | **0.68 (0.58 to 0.81)** | **<.001** | **0.75 (0.62 to 0.92)** | **0.004** | 1.32 (0.93 to 1.89) | 0.120 |
| Household income | | | | | | | | | | |
| Highest quartile | 1.00 (ref) |  | 1.00 (ref) |  | 1.00 (ref) |  | 1.00 (ref) |  | 1.00 (ref) |  |
| Third quartile | 0.94 (0.86 to 1.03) | 0.196 | **0.82 (0.71 to 0.94)** | **0.005** | **0.82 (0.70 to 0.96)** | **0.012** | 0.92 (0.78 to 1.10) | 0.370 | 1.28 (0.92 to 1.76) | 0.142 |
| Second quartile | **0.69 (0.63 to 0.75)** | **<.001** | **0.83 (0.73 to 0.96)** | **0.01** | **0.84 (0.72 to 0.98)** | **0.029** | 0.92 (0.77 to 1.09) | 0.340 | 1.33 (0.98 to 1.82) | 0.0710 |
| Lowest quartile | **0.46 (0.42 to 0.51)** | **<.001** | **0.78 (0.67 to 0.90)** | **0.001** | 0.85 (0.73 to 1.00) | 0.055 | 0.99 (0.83 to 1.19) | 0.919 | **1.55 (1.13 to 2.12)** | **0.006** |
| Smoking status | | | | | | | | | | |
| Non-smoker | 1.00 (ref) |  | 1.00 (ref) |  | 1.00 (ref) |  | 1.00 (ref) |  | 1.00 (ref) |  |
| Smoker | **1.40 (1.26 to 1.54)** | **<.001** | **1.88 (1.61 to 2.21)** | **<.001** | **2.01 (1.68 to 2.41)** | **<.001** | **1.70 (1.40 to 2.08)** | **<.001** | 0.85 (0.59 to 1.21) | 0.366 |
| Waist-to-height ratio ^b^ | | | | | | | | | | |
| Normal | 1.00 (ref) |  | 1.00 (ref) |  | 1.00 (ref) |  | 1.00 (ref) |  | 1.00 (ref) |  |
| Central adiposity | **0.76 (0.71 to 0.81)** | **<.001** | 0.98 (0.87 to 1.10) | 0.752 | 0.90 (0.78 to 1.04) | 0.141 | 0.93 (0.81 to 1.07) | 0.317 | 1.02 (0.77 to 1.35) | 0.884 |
| Daily calorie intake ^c^ | | | | | | | | | | |
| Low | 1.00 (ref) |  | 1.00 (ref) |  | 1.00 (ref) |  | 1.00 (ref) |  | 1.00 (ref) |  |
| High | 1.09 (1.01 to 1.18) | 0.034 | 1.05 (0.94 to 1.19) | 0.382 | 1.03 (0.91 to 1.18) | 0.618 | 1.08 (0.93 to 1.24) | 0.333 | 1.16 (0.91 to 1.48) | 0.221 |
| High-risk drinking ^d^ | | | | | | | | | | |
| No | 1.00 (ref) |  | 1.00 (ref) |  | 1.00 (ref) |  | 1.00 (ref) |  | 1.00 (ref) |  |
| Yes | **1.56 (1.37 to 1.79)** | **<.001** | **1.45 (1.16 to 1.83)** | **0.001** | **1.39 (1.08 to 1.80)** | **0.012** | 1.29 (1.00 to 1.67) | 0.054 | 1.07 (0.64 to 1.78) | 0.797 |

Abbreviations: BMI, body mass index; CI, confidence interval; KNHANES, Korea National Health and Nutrition Examination Survey; OR, odds ratio.

Numbers in bold indicate a significant difference (P < 0.05).

^a^ BMI was divided into four groups according to Asian-Pacific guidelines: underweight (<18.5 kg/m^2^), normal (18.5-22.9 kg/m^2^), overweight (23.0–24.9 kg/m^2^), and obese (≥25 kg/m^2^).

^b^ Waist-to-height ratio was calculated as waist circumference divided by height and categorized into two groups: normal (<0.5) and central adiposity (≥0.5).

^c^ Daily calorie intake was categorized into two groups: low (below the median) and high (above the median).

^d^ High-risk drinking was defined as consuming ≥7 drinks per occasion for men or ≥5 for women at least twice per week, classified as ‘yes’ or ‘no’ accordingly.

**Table S3.** Weighted odds ratios of dyslipidemia prevalence before and during the COVID-19 (weighted % [95% CI]) based on data obtained from the KNHANES

| Variables | | Overall (2005–2022) | | Before the pandemic (2005–2019) | | During the pandemic (2020–2022) | | Ratio of ORs (95% CI) during the pandemic compared to before the pandemic (reference) | |
| --- | --- | --- | --- | --- | --- | --- | --- | --- | --- |
|  |  | Weighted OR (95% CI) | P-value | Weighted OR (95% CI) | P-value | Weighted OR (95% CI) | P-value | Weighted ratio of ORs (95% CI) | P-value |
| Sex | Female | 1.00 (ref) |  | 1.00 (ref) |  | 1.00 (ref) |  | 1.00 (ref) |  |
|  | Male | **1.59 (1.54 to 1.65)** | **<.001** | **1.60 (1.55 to 1.66)** | **<.001** | **1.55 (1.42 to 1.69)** | **<.001** | 0.97 (0.88 to 1.06) | 0.471 |
| Age, years | 30-39 | 1.00 (ref) |  | 1.00 (ref) |  | 1.00 (ref) |  | 1.00 (ref) |  |
|  | 40-49 | **1.46 (1.38 to 1.54)** | **<.001** | **1.42 (1.34 to 1.50)** | **<.001** | **1.66 (1.45 to 1.89)** | **<.001** | **1.17 (1.02 to 1.35)** | **0.030** |
|  | 50-59 | **2.39 (2.27 to 2.52)** | **<.001** | **2.31 (2.18 to 2.45)** | **<.001** | **2.72 (2.37 to 3.12)** | **<.001** | **1.18 (1.01 to 1.37)** | **0.032** |
|  | 60-69 | **3.25 (3.07 to 3.44)** | **<.001** | **3.15 (2.97 to 3.35)** | **<.001** | **3.56 (3.09 to 4.10)** | **<.001** | 1.13 (0.97 to 1.32) | 0.127 |
|  | ≥70 | **2.96 (2.81 to 3.13)** | **<.001** | **2.88 (2.71 to 3.05)** | **<.001** | **3.30 (2.87 to 3.78)** | **<.001** | 1.15 (0.99 to 1.33) | 0.077 |
| Region of residence | Urban | 1.00 (ref) |  | 1.00 (ref) |  | 1.00 (ref) |  | 1.00 (ref) |  |
|  | Rural | **1.13 (1.08 to 1.19)** | **<.001** | **1.14 (1.09 to 1.20)** | **<.001** | **1.13 (1.01 to 1.27)** | **0.029** | 0.99 (0.88 to 1.12) | 0.921 |
| BMI group ^a^ | Underweight | 1.00 (ref) |  | 1.00 (ref) |  | 1.00 (ref) |  | 1.00 (ref) |  |
|  | Normal | **1.92 (1.71 to 2.15)** | **<.001** | **1.94 (1.71 to 2.19)** | **<.001** | **1.88 (1.43 to 2.47)** | **<.001** | 0.97 (0.72 to 1.31) | 0.845 |
|  | Overweight | **3.55 (3.16 to 3.98)** | **<.001** | **3.56 (3.14 to 4.03)** | **<.001** | **3.55 (2.69 to 4.69)** | **<.001** | 1.00 (0.74 to 1.35) | 0.988 |
|  | Obese | **5.62 (5.01 to 6.30)** | **<.001** | **5.68 (5.02 to 6.42)** | **<.001** | **5.38 (4.06 to 7.12)** | **<.001** | 0.95 (0.70 to 1.29) | 0.730 |
| Educational background | College or higher | 1.00 (ref) |  | 1.00 (ref) |  | 1.00 (ref) |  | 1.00 (ref) |  |
|  | High school | **1.22 (1.17 to 1.27)** | **<.001** | **1.19 (1.14 to 1.25)** | **<.001** | **1.42 (1.29 to 1.56)** | **<.001** | **1.19 (1.07 to 1.32)** | **0.001** |
|  | Middle school | **1.72 (1.63 to 1.82)** | **<.001** | **1.71 (1.61 to 1.82)** | **<.001** | **1.93 (1.68 to 2.20)** | **<.001** | 1.12 (0.97 to 1.30) | 0.122 |
|  | Elementary school or lower | **1.95 (1.87 to 2.04)** | **<.001** | **1.98 (1.89 to 2.08)** | **<.001** | **2.04 (1.82 to 2.28)** | **<.001** | 1.03 (0.91 to 1.17) | 0.662 |
| Household income | Highest quartile | 1.00 (ref) |  | 1.00 (ref) |  | 1.00 (ref) |  | 1.00 (ref) |  |
|  | Third quartile | 0.99 (0.95 to 1.04) | 0.646 | 0.98 (0.93 to 1.03) | 0.403 | 1.04 (0.93 to 1.16) | 0.538 | 1.06 (0.94 to 1.19) | 0.368 |
|  | Second quartile | **1.16 (1.11 to 1.22)** | **<.001** | **1.13 (1.08 to 1.19)** | **<.001** | **1.31 (1.17 to 1.47)** | **<.001** | **1.16 (1.03 to 1.31)** | **0.018** |
|  | Lowest quartile | **1.60 (1.52 to 1.68)** | **<.001** | **1.59 (1.50 to 1.67)** | **<.001** | **1.68 (1.49 to 1.90)** | **<.001** | 1.06 (0.93 to 1.21) | 0.384 |
| Smoking status | Non-smoker | 1.00 (ref) |  | 1.00 (ref) |  | 1.00 (ref) |  | 1.00 (ref) |  |
|  | Smoker | **1.46 (1.41 to 1.51)** | **<.001** | **1.47 (1.41 to 1.52)** | **<.001** | **1.44 (1.33 to 1.57)** | **<.001** | 0.98 (0.90 to 1.08) | 0.746 |
| Waist-to-height ratio ^b^ | Normal | 1.00 (ref) |  | 1.00 (ref) |  | 1.00 (ref) |  | 1.00 (ref) |  |
|  | Central adiposity | **3.14 (3.03 to 3.25)** | **<.001** | **3.11 (3.00 to 3.23)** | **<.001** | **3.18 (2.91 to 3.46)** | **<.001** | 1.02 (0.93 to 1.12) | 0.658 |
| Daily calorie intake ^c^ | Low | 1.00 (ref) |  | 1.00 (ref) |  | 1.00 (ref) |  | 1.00 (ref) |  |
|  | High | 0.98 (0.95 to 1.02) | 0.286 | 0.98 (0.95 to 1.02) | 0.421 | 0.98 (0.90 to 1.06) | 0.540 | 0.99 (0.90 to 1.09) | 0.843 |
| High-risk drinking ^d^ | No | 1.00 (ref) |  | 1.00 (ref) |  | 1.00 (ref) |  | 1.00 (ref) |  |
|  | Yes | **1.18 (1.12 to 1.25)** | **<.001** | **1.17 (1.10 to 1.24)** | **<.001** | **1.24 (1.09 to 1.40)** | **0.001** | 1.06 (0.92 to 1.22) | 0.434 |

Abbreviations: BMI, body mass index; CI, confidence interval; KNHANES, Korea National Health and Nutrition Examination Survey; OR, odds ratio.

The numbers in bold indicate significant differences (P < 0.05).

^a^ BMI was divided into four groups according to Asian-Pacific guidelines: underweight (<18.5 kg/m^2^), normal (18.5-22.9 kg/m^2^), overweight (23.0–24.9 kg/m^2^), and obese (≥25 kg/m^2^).

^b^ Waist-to-height ratio was calculated as waist circumference divided by height and categorized into two groups: normal (<0.5) and central adiposity (≥0.5).

^c^ Daily calorie intake was categorized into two groups: low (below the median) and high (above the median).

^d^ High-risk drinking was defined as consuming ≥7 drinks per occasion for men or ≥5 for women at least twice per week, classified as ‘yes’ or ‘no’ accordingly.

**Table S4.** Weighted odds ratios of awareness of dyslipidemia before and during the COVID-19 (weighted % [95% CI]) based on data obtained from the KNHANES

| Variables | | Overall (2005–2022) | | Before the pandemic (2005–2019) | | During the pandemic (2020–2022) | | Ratio of ORs (95% CI) during the pandemic compared to before the pandemic (reference) | |
| --- | --- | --- | --- | --- | --- | --- | --- | --- | --- |
|  |  | Weighted OR (95% CI) | P-value | Weighted OR (95% CI) | P-value | Weighted OR (95% CI) | P-value | Weighted ratio of ORs (95% CI) | P-value |
| Sex | Female | 1.00 (ref) |  | 1.00 (ref) |  | 1.00 (ref) |  | 1.00 (ref) |  |
|  | Male | **0.51 (0.48 to 0.54)** | **<.001** | **0.51 (0.48 to 0.54)** | **<.001** | **0.48 (0.43 to 0.54)** | **<.001** | 0.95 (0.84 to 1.08) | 0.470 |
| Age, years | 30-39 | 1.00 (ref) |  | 1.00 (ref) |  | 1.00 (ref) |  | 1.00 (ref) |  |
|  | 40-49 | **2.62 (2.29 to 2.99)** | **<.001** | **2.35 (2.02 to 2.73)** | **<.001** | **3.60 (2.66 to 4.86)** | **<.001** | **1.53 (1.09 to 2.15)** | **0.013** |
|  | 50-59 | **5.84 (5.15 to 6.63)** | **<.001** | **5.30 (4.61 to 6.09)** | **<.001** | **7.64 (5.69 to 10.26)** | **<.001** | **1.44 (1.04 to 2.00)** | **0.028** |
|  | 60-69 | **10.00 (8.80 to 11.36)** | **<.001** | **8.66 (7.52 to 9.96)** | **<.001** | **14.17 (10.49 to 19.15)** | **<.001** | **1.64 (1.17 to 2.28)** | **0.004** |
|  | ≥70 | **8.35 (7.34 to 9.50)** | **<.001** | **6.32 (5.47 to 7.30)** | **<.001** | **19.93 (14.81 to 26.81)** | **<.001** | **3.15 (2.27 to 4.38)** | **<.001** |
| Region of residence | Urban | 1.00 (ref) |  | 1.00 (ref) |  | 1.00 (ref) |  | 1.00 (ref) |  |
|  | Rural | **0.87 (0.81 to 0.95)** | **0.001** | **0.86 (0.78 to 0.94)** | **0.001** | 1.01 (0.87 to 1.18) | 0.858 | 1.18 (0.99 to 1.41) | 0.060 |
| BMI group ^a^ | Underweight | 1.00 (ref) |  | 1.00 (ref) |  | 1.00 (ref) |  | 1.00 (ref) |  |
|  | Normal | **1.37 (1.09 to 1.73)** | **0.007** | **1.63 (1.25 to 2.11)** | **<.001** | 1.04 (0.66 to 1.64) | 0.872 | 0.64 (0.38 to 1.08) | 0.095 |
|  | Overweight | **1.47 (1.16 to 1.85)** | **0.001** | **1.76 (1.35 to 2.29)** | **<.001** | 1.05 (0.66 to 1.68) | 0.832 | 0.60 (0.35 to 1.02) | 0.061 |
|  | Obese | **1.49 (1.18 to 1.88)** | **0.001** | **1.83 (1.41 to 2.38)** | **<.001** | 0.92 (0.58 to 1.47) | 0.727 | **0.50 (0.30 to 0.85)** | **0.011** |
| Educational background | College or higher | 1.00 (ref) |  | 1.00 (ref) |  | 1.00 (ref) |  | 1.00 (ref) |  |
|  | High school | **1.41 (1.32 to 1.51)** | **<.001** | **1.37 (1.27 to 1.48)** | **<.001** | **1.76 (1.54 to 2.01)** | **<.001** | **1.28 (1.10 to 1.50)** | **0.002** |
|  | Middle school | **2.18 (2.01 to 2.37)** | **<.001** | **2.19 (1.99 to 2.40)** | **<.001** | **3.27 (2.70 to 3.96)** | **<.001** | **1.50 (1.21 to 1.85)** | **<.001** |
|  | Elementary school or lower | **1.98 (1.84 to 2.13)** | **<.001** | **2.00 (1.84 to 2.17)** | **<.001** | **3.62 (3.06 to 4.28)** | **<.001** | **1.81 (1.50 to 2.18)** | **<.001** |
| Household income | Highest quartile | 1.00 (ref) |  | 1.00 (ref) |  | 1.00 (ref) |  | 1.00 (ref) |  |
|  | Third quartile | 1.00 (0.93 to 1.08) | 0.931 | 0.98 (0.90 to 1.07) | 0.593 | 1.11 (0.94 to 1.30) | 0.214 | 1.13 (0.94 to 1.35) | 0.181 |
|  | Second quartile | **1.13 (1.05 to 1.21)** | **0.002** | **1.11 (1.02 to 1.20)** | **0.017** | **1.29 (1.10 to 1.52)** | **0.002** | 1.17 (0.98 to 1.40) | 0.090 |
|  | Lowest quartile | **1.47 (1.36 to 1.59)** | **<.001** | **1.41 (1.29 to 1.53)** | **<.001** | **2.08 (1.75 to 2.48)** | **<.001** | **1.48 (1.22 to 1.80)** | **<.001** |
| Smoking status | Non-smoker | 1.00 (ref) |  | 1.00 (ref) |  | 1.00 (ref) |  | 1.00 (ref) |  |
|  | Smoker | **0.59 (0.56 to 0.62)** | **<.001** | **0.58 (0.55 to 0.62)** | **<.001** | **0.61 (0.54 to 0.68)** | **<.001** | 1.04 (0.91 to 1.18) | 0.552 |
| Waist-to-height ratio ^b^ | Normal | 1.00 (ref) |  | 1.00 (ref) |  | 1.00 (ref) |  | 1.00 (ref) |  |
|  | Central adiposity | **1.59 (1.50 to 1.68)** | **<.001** | **1.61 (1.51 to 1.73)** | **<.001** | **1.37 (1.21 to 1.55)** | **<.001** | **0.85 (0.74 to 0.98)** | **0.021** |
| Daily calorie intake ^c^ | Low | 1.00 (ref) |  | 1.00 (ref) |  | 1.00 (ref) |  | 1.00 (ref) |  |
|  | High | **0.70 (0.67 to 0.74)** | **<.001** | **0.71 (0.67 to 0.75)** | **<.001** | **0.69 (0.61 to 0.78)** | **<.001** | 0.97 (0.84 to 1.11) | 0.656 |
| High-risk drinking ^d^ | No | 1.00 (ref) |  | 1.00 (ref) |  | 1.00 (ref) |  | 1.00 (ref) |  |
|  | Yes | **0.67 (0.61 to 0.72)** | **<.001** | **0.70 (0.64 to 0.77)** | **<.001** | **0.55 (0.47 to 0.65)** | **<.001** | **0.79 (0.65 to 0.95)** | **0.014** |

Abbreviations: BMI, body mass index; CI, confidence interval; KNHANES, Korea National Health and Nutrition Examination Survey; OR, odds ratio.

The numbers in bold indicate significant differences (P < 0.05).

^a^ BMI was divided into four groups according to Asian-Pacific guidelines: underweight (<18.5 kg/m^2^), normal (18.5-22.9 kg/m^2^), overweight (23.0–24.9 kg/m^2^), and obese (≥25 kg/m^2^).

^b^ Waist-to-height ratio was calculated as waist circumference divided by height and categorized into two groups: normal (<0.5) and central adiposity (≥0.5).

^c^ Daily calorie intake was categorized into two groups: low (below the median) and high (above the median).

^d^ High-risk drinking was defined as consuming ≥7 drinks per occasion for men or ≥5 for women at least twice per week, classified as ‘yes’ or ‘no’ accordingly.

**Table S5.** Weighted odds ratios of treatment of dyslipidemia before and during the COVID-19 (weighted % [95% CI]) based on data obtained from the KNHANES

| Variables | | Overall (2005–2022) | | Before the pandemic (2005–2019) | | During the pandemic (2020–2022) | | Ratio of ORs (95% CI) during the pandemic compared to before the pandemic (reference) | |
| --- | --- | --- | --- | --- | --- | --- | --- | --- | --- |
|  |  | Weighted OR (95% CI) | P-value | Weighted OR (95% CI) | P-value | Weighted OR (95% CI) | P-value | Weighted ratio of ORs (95% CI) | P-value |
| Sex | Female | 1.00 (ref) |  | 1.00 (ref) |  | 1.00 (ref) |  | 1.00 (ref) |  |
|  | Male | **0.51 (0.48 to 0.54)** | **<.001** | **0.49 (0.46 to 0.52)** | **<.001** | **0.50 (0.45 to 0.56)** | **<.001** | 1.03 (0.90 to 1.17) | 0.676 |
| Age, years | 30-39 | 1.00 (ref) |  | 1.00 (ref) |  | 1.00 (ref) |  | 1.00 (ref) |  |
|  | 40-49 | **3.69 (2.98 to 4.56)** | **<.001** | **3.48 (2.70 to 4.49)** | **<.001** | **3.95 (2.67 to 5.83)** | **<.001** | 1.13 (0.71 to 1.81) | 0.598 |
|  | 50-59 | **9.53 (7.80 to 11.64)** | **<.001** | **9.70 (7.67 to 12.27)** | **<.001** | **8.66 (5.97 to 12.56)** | **<.001** | 0.89 (0.57 to 1.39) | 0.612 |
|  | 60-69 | **18.62 (15.28 to 22.68)** | **<.001** | **18.70 (14.84 to 23.56)** | **<.001** | **16.61 (11.42 to 24.17)** | **<.001** | 0.89 (0.57 to 1.38) | 0.598 |
|  | ≥70 | **18.36 (15.03 to 22.44)** | **<.001** | **16.39 (12.95 to 20.74)** | **<.001** | **24.64 (17.00 to 35.71)** | **<.001** | 1.50 (0.97 to 2.33) | 0.069 |
| Region of residence | Urban | 1.00 (ref) |  | 1.00 (ref) |  | 1.00 (ref) |  | 1.00 (ref) |  |
|  | Rural | **0.89 (0.81 to 0.98)** | **0.018** | **0.86 (0.77 to 0.96)** | **0.009** | 1.06 (0.90 to 1.24) | 0.513 | **1.22 (1.01 to 1.48)** | **0.042** |
| BMI group ^a^ | Underweight | 1.00 (ref) |  | 1.00 (ref) |  | 1.00 (ref) |  | 1.00 (ref) |  |
|  | Normal | **1.41 (1.07 to 1.85)** | **0.015** | **1.79 (1.30 to 2.46)** | **<.001** | 1.09 (0.68 to 1.76) | 0.726 | 0.61 (0.34 to 1.08) | 0.092 |
|  | Overweight | **1.53 (1.16 to 2.02)** | **0.003** | **1.94 (1.40 to 2.67)** | **<.001** | 1.18 (0.73 to 1.92) | 0.506 | 0.61 (0.34 to 1.09) | 0.096 |
|  | Obese | **1.62 (1.23 to 2.14)** | **0.001** | **2.17 (1.58 to 2.98)** | **<.001** | 1.03 (0.63 to 1.68) | 0.901 | **0.48 (0.27 to 0.85)** | **0.013** |
| Educational background | College or higher | 1.00 (ref) |  | 1.00 (ref) |  | 1.00 (ref) |  | 1.00 (ref) |  |
|  | High school | **1.49 (1.37 to 1.62)** | **<.001** | **1.51 (1.36 to 1.67)** | **<.001** | **1.75 (1.50 to 2.04)** | **<.001** | 1.16 (0.97 to 1.39) | 0.113 |
|  | Middle school | **2.32 (2.12 to 2.55)** | **<.001** | **2.50 (2.23 to 2.79)** | **<.001** | **3.22 (2.67 to 3.88)** | **<.001** | **1.29 (1.04 to 1.60)** | **0.023** |
|  | Elementary school or lower | **2.36 (2.16 to 2.57)** | **<.001** | **2.58 (2.33 to 2.86)** | **<.001** | **3.88 (3.29 to 4.58)** | **<.001** | **1.50 (1.24 to 1.82)** | **<.001** |
| Household income | Highest quartile | 1.00 (ref) |  | 1.00 (ref) |  | 1.00 (ref) |  | 1.00 (ref) |  |
|  | Third quartile | 1.04 (0.95 to 1.14) | 0.384 | 1.04 (0.94 to 1.16) | 0.432 | 1.07 (0.90 to 1.27) | 0.432 | 1.03 (0.84 to 1.26) | 0.789 |
|  | Second quartile | **1.25 (1.14 to 1.36)** | **<.001** | **1.24 (1.12 to 1.36)** | **<.001** | **1.43 (1.21 to 1.68)** | **<.001** | 1.15 (0.95 to 1.40) | 0.141 |
|  | Lowest quartile | **1.69 (1.55 to 1.85)** | **<.001** | **1.72 (1.56 to 1.90)** | **<.001** | **2.09 (1.75 to 2.48)** | **<.001** | 1.21 (0.99 to 1.48) | 0.058 |
| Smoking status | Non-smoker | 1.00 (ref) |  | 1.00 (ref) |  | 1.00 (ref) |  | 1.00 (ref) |  |
|  | Smoker | **0.59 (0.56 to 0.63)** | **<.001** | **0.58 (0.54 to 0.62)** | **<.001** | **0.61 (0.54 to 0.68)** | **<.001** | 1.04 (0.92 to 1.19) | 0.515 |
| Waist-to-height ratio ^b^ | Normal | 1.00 (ref) |  | 1.00 (ref) |  | 1.00 (ref) |  | 1.00 (ref) |  |
|  | Central adiposity | **0.67 (0.61 to 0.72)** | **<.001** | **0.70 (0.64 to 0.77)** | **<.001** | **0.55 (0.47 to 0.65)** | **<.001** | **0.79 (0.65 to 0.95)** | **0.014** |
| Daily calorie intake ^c^ | Low | 1.00 (ref) |  | 1.00 (ref) |  | 1.00 (ref) |  | 1.00 (ref) |  |
|  | High | **0.64 (0.60 to 0.68)** | **<.001** | **0.63 (0.59 to 0.68)** | **<.001** | **0.65 (0.57 to 0.74)** | **<.001** | 1.03 (0.89 to 1.19) | 0.673 |
| High-risk drinking ^d^ | No | 1.00 (ref) |  | 1.00 (ref) |  | 1.00 (ref) |  | 1.00 (ref) |  |
|  | Yes | **0.59 (0.53 to 0.65)** | **<.001** | **0.58 (0.52 to 0.66)** | **<.001** | **0.55 (0.46 to 0.65)** | **<.001** | 0.94 (0.76 to 1.17) | 0.590 |

Abbreviations: BMI, body mass index; CI, confidence interval; KNHANES, Korea National Health and Nutrition Examination Survey; OR, odds ratio.

The numbers in bold indicate significant differences (P < 0.05).

^a^ BMI was divided into four groups according to Asian-Pacific guidelines: underweight (<18.5 kg/m^2^), normal (18.5-22.9 kg/m^2^), overweight (23.0–24.9 kg/m^2^), and obese (≥25 kg/m^2^).

^b^ Waist-to-height ratio was calculated as waist circumference divided by height and categorized into two groups: normal (<0.5) and central adiposity (≥0.5).

^c^ Daily calorie intake was categorized into two groups: low (below the median) and high (above the median).

^d^ High-risk drinking was defined as consuming ≥7 drinks per occasion for men or ≥5 for women at least twice per week, classified as ‘yes’ or ‘no’ accordingly.

**Table S6.** Weighted odds ratios of control among dyslipidemia before and during the COVID-19 (weighted % [95% CI]) based on data obtained from the KNHANES

| Variables | | Overall (2005–2022) | | Before the pandemic (2005–2019) | | During the pandemic (2020–2022) | | Ratio of ORs (95% CI)  during the pandemic compared to before the pandemic (reference) | |
| --- | --- | --- | --- | --- | --- | --- | --- | --- | --- |
|  |  | Weighted OR (95% CI) | P-value | Weighted OR (95% CI) | P-value | Weighted OR (95% CI) | P-value | Weighted ratio of ORs (95% CI) | P-value |
| Sex | Female | 1.00 (ref) |  | 1.00 (ref) |  | 1.00 (ref) |  | 1.00 (ref) |  |
|  | Male | **0.40 (0.38 to 0.43)** | **<.001** | **0.39 (0.36 to 0.42)** | **<.001** | **0.38 (0.34 to 0.43)** | **<.001** | 0.97 (0.85 to 1.12) | 0.714 |
| Age, years | 30-39 | 1.00 (ref) |  | 1.00 (ref) |  | 1.00 (ref) |  | 1.00 (ref) |  |
|  | 40-49 | **2.78 (2.27 to 3.41)** | **<.001** | **2.51 (2.00 to 3.17)** | **<.001** | **3.34 (2.19 to 5.09)** | **<.001** | 1.33 (0.82 to 2.15) | 0.247 |
|  | 50-59 | **6.23 (5.16 to 7.52)** | **<.001** | **5.82 (4.72 to 7.17)** | **<.001** | **6.80 (4.52 to 10.23)** | **<.001** | 1.17 (0.74 to 1.85) | 0.508 |
|  | 60-69 | **11.92 (9.89 to 14.37)** | **<.001** | **10.64 (8.63 to 13.12)** | **<.001** | **13.59 (9.06 to 20.40)** | **<.001** | 1.28 (0.81 to 2.02) | 0.294 |
|  | ≥70 | **11.11 (9.22 to 13.40)** | **<.001** | **8.95 (7.23 to 11.06)** | **<.001** | **17.35 (11.67 to 25.79)** | **<.001** | **1.94 (1.24 to 3.04)** | **0.004** |
| Region of residence | Urban | 1.00 (ref) |  | 1.00 (ref) |  | 1.00 (ref) |  | 1.00 (ref) |  |
|  | Rural | **0.85 (0.77 to 0.94)** | **0.002** | **0.84 (0.75 to 0.94)** | **0.002** | 0.97 (0.82 to 1.14) | 0.680 | 1.16 (0.95 to 1.41) | 0.157 |
| BMI group ^a^ | Underweight | 1.00 (ref) |  | 1.00 (ref) |  | 1.00 (ref) |  | 1.00 (ref) |  |
|  | Normal | 1.08 (0.83 to 1.41) | 0.551 | 1.30 (0.96 to 1.75) | 0.090 | 0.83 (0.51 to 1.34) | 0.437 | 0.64 (0.36 to 1.13) | 0.121 |
|  | Overweight | 0.99 (0.76 to 1.29) | 0.951 | 1.16 (0.86 to 1.58) | 0.336 | 0.77 (0.47 to 1.25) | 0.283 | 0.66 (0.37 to 1.17) | 0.156 |
|  | Obese | 0.87 (0.67 to 1.14) | 0.312 | 1.09 (0.81 to 1.47) | 0.580 | **0.54 (0.33 to 0.88)** | **0.014** | **0.50 (0.28 to 0.88)** | **0.017** |
| Educational background | College or higher | 1.00 (ref) |  | 1.00 (ref) |  | 1.00 (ref) |  | 1.00 (ref) |  |
|  | High school | **1.40 (1.28 to 1.52)** | **<.001** | **1.43 (1.29 to 1.59)** | **<.001** | **1.56 (1.34 to 1.82)** | **<.001** | 1.09 (0.91 to 1.32) | 0.343 |
|  | Middle school | **2.11 (1.91 to 2.33)** | **<.001** | **2.22 (1.97 to 2.49)** | **<.001** | **2.89 (2.38 to 3.50)** | **<.001** | **1.30 (1.04 to 1.63)** | **0.021** |
|  | Elementary school or lower | **1.96 (1.79 to 2.14)** | **<.001** | **2.09 (1.88 to 2.32)** | **<.001** | **3.11 (2.63 to 3.69)** | **<.001** | **1.49 (1.22 to 1.82)** | **<.001** |
| Household income | Highest quartile | 1.00 (ref) |  | 1.00 (ref) |  | 1.00 (ref) |  | 1.00 (ref) |  |
|  | Third quartile | 1.01 (0.92 to 1.12) | 0.770 | 0.98 (0.87 to 1.09) | 0.663 | 1.13 (0.94 to 1.35) | 0.196 | 1.15 (0.93 to 1.42) | 0.186 |
|  | Second quartile | **1.17 (1.07 to 1.29)** | **0.001** | **1.16 (1.05 to 1.29)** | **0.006** | **1.33 (1.11 to 1.59)** | **0.002** | 1.14 (0.93 to 1.40) | 0.209 |
|  | Lowest quartile | **1.45 (1.32 to 1.59)** | **<.001** | **1.40 (1.26 to 1.56)** | **<.001** | **1.90 (1.60 to 2.27)** | **<.001** | **1.36 (1.10 to 1.66)** | **0.004** |
| Smoking status | Non-smoker | 1.00 (ref) |  | 1.00 (ref) |  | 1.00 (ref) |  | 1.00 (ref) |  |
|  | Smoker | **0.47 (0.44 to 0.50)** | **<.001** | **0.45 (0.42 to 0.49)** | **<.001** | **0.47 (0.41 to 0.53)** | **<.001** | 1.03 (0.89 to 1.19) | 0.680 |
| Waist-to-height ratio ^b^ | Normal | 1.00 (ref) |  | 1.00 (ref) |  | 1.00 (ref) |  | 1.00 (ref) |  |
|  | Central adiposity | **1.24 (1.16 to 1.33)** | **<.001** | **1.23 (1.14 to 1.34)** | **<.001** | 1.09 (0.96 to 1.25) | 0.185 | 0.89 (0.76 to 1.04) | 0.132 |
| Daily calorie intake ^c^ | Low | 1.00 (ref) |  | 1.00 (ref) |  | 1.00 (ref) |  | 1.00 (ref) |  |
|  | High | **0.67 (0.63 to 0.72)** | **<.001** | **0.68 (0.63 to 0.73)** | **<.001** | **0.66 (0.58 to 0.75)** | **<.001** | 0.97 (0.84 to 1.13) | 0.741 |
| High-risk drinking ^d^ | No | 1.00 (ref) |  | 1.00 (ref) |  | 1.00 (ref) |  | 1.00 (ref) |  |
|  | Yes | **0.55 (0.50 to 0.62)** | **<.001** | **0.58 (0.51 to 0.66)** | **<.001** | **0.48 (0.39 to 0.58)** | **<.001** | 0.83 (0.65 to 1.05) | 0.115 |

Abbreviations: BMI, body mass index; CI, confidence interval; KNHANES, Korea National Health and Nutrition Examination Survey; OR, odds ratio.

The numbers in bold indicate significant differences (P < 0.05).

^a^ BMI was divided into four groups according to Asian-Pacific guidelines: underweight (<18.5 kg/m^2^), normal (18.5-22.9 kg/m^2^), overweight (23.0–24.9 kg/m^2^), and obese (≥25 kg/m^2^).

^b^ Waist-to-height ratio was calculated as waist circumference divided by height and categorized into two groups: normal (<0.5) and central adiposity (≥0.5).

^c^ Daily calorie intake was categorized into two groups: low (below the median) and high (above the median).

^d^ High-risk drinking was defined as consuming ≥7 drinks per occasion for men or ≥5 for women at least twice per week, classified as ‘yes’ or ‘no’ accordingly.

**Table S7.** Weighted odds ratios of dyslipidemia control among treatment before and during COVID-19 (weighted % [95% CI]) based on data obtained from KNHANES

| Variables | | Overall (2005–2022) | | Before the pandemic (2005–2019) | | During the pandemic (2020–2022) | | Ratio of ORs (95% CI) during the pandemic compared to before the pandemic (reference) | |
| --- | --- | --- | --- | --- | --- | --- | --- | --- | --- |
|  |  | Weighted OR (95% CI) | P-value | Weighted OR (95% CI) | P-value | Weighted OR (95% CI) | P-value | Weighted ratio of ORs (95% CI) | P-value |
| Sex | Female | 1.00 (ref) |  | 1.00 (ref) |  | 1.00 (ref) |  | 1.00 (ref) |  |
|  | Male | **0.48 (0.43 to 0.54)** | **<.001** | **0.51 (0.44 to 0.58)** | **<.001** | **0.40 (0.32 to 0.50)** | **<.001** | 0.79 (0.61 to 1.02) | 0.070 |
| Age, years | 30-39 | 1.00 (ref) |  | 1.00 (ref) |  | 1.00 (ref) |  | 1.00 (ref) |  |
|  | 40-49 | **1.68 (1.09 to 2.59)** | **0.018** | **1.80 (1.07 to 3.01)** | **0.026** | 1.37 (0.61 to 3.08) | 0.447 | 0.76 (0.29 to 1.99) | 0.580 |
|  | 50-59 | **1.90 (1.27 to 2.85)** | **0.002** | **1.97 (1.23 to 3.18)** | **0.005** | 1.77 (0.81 to 3.83) | 0.150 | 0.90 (0.36 to 2.22) | 0.811 |
|  | 60-69 | **2.40 (1.62 to 3.56)** | **<.001** | **2.43 (1.52 to 3.88)** | **<.001** | **2.28 (1.09 to 4.79)** | **0.029** | 0.94 (0.39 to 2.26) | 0.893 |
|  | ≥70 | **2.62 (1.76 to 3.89)** | **<.001** | **2.74 (1.71 to 4.40)** | **<.001** | **2.21 (1.04 to 4.68)** | **0.039** | 0.81 (0.33 to 1.96) | 0.635 |
| Region of residence | Urban | 1.00 (ref) |  | 1.00 (ref) |  | 1.00 (ref) |  | 1.00 (ref) |  |
|  | Rural | **0.85 (0.74 to 0.98)** | **0.026** | 0.88 (0.74 to 1.05) | 0.151 | 0.80 (0.63 to 1.01) | 0.065 | 0.91 (0.68 to 1.22) | 0.534 |
| BMI group ^a^ | Underweight | 1.00 (ref) |  | 1.00 (ref) |  | 1.00 (ref) |  | 1.00 (ref) |  |
|  | Normal | 0.90 (0.49 to 1.66) | 0.746 | 1.11 (0.55 to 2.22) | 0.774 | 0.80 (0.26 to 2.45) | 0.697 | 0.72 (0.19 to 2.71) | 0.631 |
|  | Overweight | 0.63 (0.34 to 1.15) | 0.133 | 0.73 (0.36 to 1.48) | 0.385 | 0.60 (0.20 to 1.81) | 0.358 | 0.81 (0.22 to 3.02) | 0.756 |
|  | Obese | **0.44 (0.24 to 0.81)** | **0.008** | 0.59 (0.29 to 1.20) | 0.145 | **0.31 (0.10 to 0.93)** | **0.036** | 0.52 (0.14 to 1.92) | 0.327 |
| Educational background | College or higher | 1.00 (ref) |  | 1.00 (ref) |  | 1.00 (ref) |  | 1.00 (ref) |  |
|  | High school | 1.01 (0.86 to 1.18) | 0.914 | 1.07 (0.89 to 1.30) | 0.461 | 0.97 (0.74 to 1.27) | 0.804 | 0.90 (0.65 to 1.25) | 0.530 |
|  | Middle school | 1.02 (0.86 to 1.22) | 0.829 | 1.08 (0.88 to 1.34) | 0.456 | 1.10 (0.80 to 1.52) | 0.552 | 1.02 (0.69 to 1.49) | 0.936 |
|  | Elementary school or lower | 0.95 (0.82 to 1.11) | 0.545 | 1.01 (0.84 to 1.21) | 0.909 | 1.11 (0.83 to 1.48) | 0.478 | 1.10 (0.78 to 1.54) | 0.592 |
| Household income | Highest quartile | 1.00 (ref) |  | 1.00 (ref) |  | 1.00 (ref) |  | 1.00 (ref) |  |
|  | Third quartile | 1.07 (0.91 to 1.27) | 0.393 | 1.03 (0.85 to 1.26) | 0.741 | 1.17 (0.87 to 1.56) | 0.297 | 1.13 (0.79 to 1.60) | 0.502 |
|  | Second quartile | 1.09 (0.93 to 1.27) | 0.305 | 1.10 (0.91 to 1.32) | 0.335 | 1.10 (0.83 to 1.46) | 0.517 | 1.00 (0.71 to 1.41) | 0.996 |
|  | Lowest quartile | 0.95 (0.81 to 1.11) | 0.494 | 0.92 (0.76 to 1.10) | 0.364 | 1.10 (0.83 to 1.47) | 0.514 | 1.20 (0.85 to 1.68) | 0.298 |
| Smoking status | Non-smoker | 1.00 (ref) |  | 1.00 (ref) |  | 1.00 (ref) |  | 1.00 (ref) |  |
|  | Smoker | **0.48 (0.43 to 0.54)** | **<.001** | **0.50 (0.44 to 0.57)** | **<.001** | **0.43 (0.34 to 0.53)** | **<.001** | 0.85 (0.66 to 1.10) | 0.212 |
| Waist-to-height ratio ^b^ | Normal | 1.00 (ref) |  | 1.00 (ref) |  | 1.00 (ref) |  | 1.00 (ref) |  |
|  | Central adiposity | **0.56 (0.49 to 0.65)** | **<.001** | **0.59 (0.50 to 0.70)** | **<.001** | **0.48 (0.36 to 0.64)** | **<.001** | 0.81 (0.58 to 1.12) | 0.207 |
| Daily calorie intake ^c^ | Low | 1.00 (ref) |  | 1.00 (ref) |  | 1.00 (ref) |  | 1.00 (ref) |  |
|  | High | **0.84 (0.74 to 0.94)** | **0.003** | 0.88 (0.76 to 1.01) | 0.068 | **0.72 (0.57 to 0.90)** | **0.004** | 0.82 (0.63 to 1.07) | 0.136 |
| High-risk drinking ^d^ | No | 1.00 (ref) |  | 1.00 (ref) |  | 1.00 (ref) |  | 1.00 (ref) |  |
|  | Yes | **0.60 (0.50 to 0.73)** | **<.001** | **0.63 (0.50 to 0.80)** | **<.001** | **0.53 (0.38 to 0.72)** | **<.001** | 0.83 (0.56 to 1.24) | 0.363 |

Abbreviations: BMI, body mass index; CI, confidence interval; KNHANES, Korea National Health and Nutrition Examination Survey; OR, odds ratio.

The numbers in bold indicate significant differences (P < 0.05).

^a^ BMI was divided into four groups according to Asian-Pacific guidelines: underweight (<18.5 kg/m^2^), normal (18.5-22.9 kg/m^2^), overweight (23.0–24.9 kg/m^2^), and obese (≥25 kg/m^2^).

^b^ Waist-to-height ratio was calculated as waist circumference divided by height and categorized into two groups: normal (<0.5) and central adiposity (≥0.5).

^c^ Daily calorie intake was categorized into two groups: low (below the median) and high (above the median).

^d^ High-risk drinking was defined as consuming ≥7 drinks per occasion for men or ≥5 for women at least twice per week, classified as ‘yes’ or ‘no’ accordingly.
